# Prokaryotic morphological features and maintenance activities governed by seasonal productivity conditions

**DOI:** 10.1093/femsec/fiae121

**Published:** 2024-09-11

**Authors:** Ashish Verma, Dennis Amnebrink, Cheng Choo Lee, Sun Nyunt Wai, Linda Sandblad, Jarone Pinhassi, Johan Wikner

**Affiliations:** Department of Ecology and Environmental Science, Umeå University, SE-901 87 Umeå, Sweden; Umeå Marine Sciences Centre, Norrbyn 557, SE-905 71 Hörnefors, Sweden; Centre for Ecology and Evolution in Microbial Model Systems – EEMiS, Linnaeus University, SE-391 82 Kalmar, Sweden; Umeå Centre for Electron Microscopy, Department of Chemistry, Umeå University, SE-901 87 Umeå, Sweden; Department of Molecular Biology, Umeå University, SE-901 87 Umeå, Sweden; Umeå Centre for Electron Microscopy, Department of Chemistry, Umeå University, SE-901 87 Umeå, Sweden; Centre for Ecology and Evolution in Microbial Model Systems – EEMiS, Linnaeus University, SE-391 82 Kalmar, Sweden; Department of Ecology and Environmental Science, Umeå University, SE-901 87 Umeå, Sweden; Umeå Marine Sciences Centre, Norrbyn 557, SE-905 71 Hörnefors, Sweden

**Keywords:** cell shape, maintenance activities, mesocosm, morphology, prokaryotes, respiration

## Abstract

Prokaryotic maintenance respiration and associated metabolic activities constitute a considerable proportion of the total respiration of carbon to CO_2_ in the ocean’s mixed layer. However, seasonal influences on prokaryotic maintenance activities in terms of morphological and metabolic adaptations at low (winter) and high productivity (summer) are still unclear. To address this, we examined the natural prokaryotic communities at the mesocosm scale to analyse the differences in their morphological features and gene expression at low and high maintenance respiration, experimentally manipulated with the specific growth rate. Here, we showed that morphological features including membrane blebbing, membrane vesicles, and cell‒cell connections occurred under high productivity. Metabolic adaptations associated with maintenance activities were observed under low productivity. Several Kyoto Encyclopedia of Genes and Genomes categories related to signal transduction, energy metabolism, and translational machinery supported maintenance activities under simulated winter conditions. Differential abundances of genes related to transporters, osmoregulation, nitrogen metabolism, ribosome biogenesis, and cold stress were observed. Our results demonstrate how specific growth rate in different seasons can influence resource allocation at the levels of morphological features and metabolic adaptations. This motivates further study of morphological features and their ecological role during high productivity, while investigations of metabolic adaptations during low productivity can advance our knowledge about maintenance activities.

## Teaser

Seasonal variations influence prokaryotic morphology and maintenance metabolism in marine ecosystems.

## Introduction

A variety of prokaryotic cell shapes, sizes, and aggregates exist in different biomes in the biosphere (Starr et al. 2013). The selective pressure of the environment on prokaryotes leads to a limited set of morphologies influenced by primary (nutrient acquisitions, cell division, and predators) and secondary mechanisms (attachment to surfaces, passive dispersal, active motility, and internal/external differentiation) (Justice et al. 2004). Despite the increased knowledge of prokaryotic community compositions in different marine ecosystems through sequencing-based projects (Young 2007, Karsenti et al. 2011, Sunagawa et al. 2020), there is a paucity of information on the distribution of cell shape and morphological features in natural prokaryotic communities in response to environmental factors. However, axenic cultures from different ecosystems exhibit more than 19 different cell shapes, with the majority belonging to rods, cocci, ovoids, and spirals (https://bacdive.dsmz.de/dashboard-type-strains) (Arnosti 2011, Reimer et al. 2019). Prokaryotes exist in complex interactions with both living (protists, protozoa, phyto-and zooplankton) and nonliving (dissolved organic matter, particulate organic matter, and inorganic matter) factors in natural ecosystems (Pollet et al. 2018, Heinrichs et al. 2020); thus, microscopic analysis of natural communities can reveal *in situ* morphological complexity.

The morphological complexities have a significant impact on the survival and fitness of the prokaryotic communities in the natural ecosystems. The reports of prokaryotic morphological features from the natural communities are rare, however the features including membrane blebbing, membrane vesicles, cell–cell connections, extracellular polymeric substances (EPS), pili, and flagella are well known from culture-dependent studies (Wagner and Brun 2007, Adams and Errington 2009, Ellison et al. 2018, Troselj et al. 2018, Zhao et al. 2018, Nagakubo et al. 2019, Flemming et al. 2023). Although membrane blebs (protrusion of inner/outer cell membrane) are commonly recognized as a hallmark of apoptosis, they also serve significant functions in normal cell processes such as cell spreading (Norman et al. 2011), and regulating the movement of migrating cells (Fackler and Grosse 2008, Bergert et al. 2012). The classical membrane vesicles (extracellularly spherical cells; ∼50–250 nm in diameter), commonly known as outer membrane vesicles, originate from outer membrane blebbing in Gram-negative bacteria (Schwechheimer et al. 2015, Jan 2017). The release of membrane vesicles by prokaryotes mediates several functions, such as horizontal gene transfer, biofilm formation, quorum sensing, virulence, seaweed cell wall degradation, cellular defence, the transfer of organic carbon, and the secretion of misfolded proteins (Mashburn and Whiteley 2005, McBroom and Kuehn 2007, Yonezawa et al. 2009, Kulp and Kuehn 2010, Biller et al. 2014, Tashiro et al. 2017, Naval and Chandra 2019, Mozaheb and Mingeot-Leclercq 2020). The interaction by cell‒cell connections can be either positive or negative depending upon the ability of the community members to either inhibit or promote the growth of another species in response to nutrient availability (Bruno et al. 2003, Ghoul and Mitri 2016). EPS secreted by marine prokaryotes can be in the form of proteins, polysaccharides, lipids, and nucleic acids, which can influence adherence, biofilm formation, cell‒cell recognition, nutrient sequestration, and protection against toxins (Zhang et al. 2015, Decho and Gutierrez 2017). Pili play important roles in twitching motility, surface attachment, biofilm formation, cell‒cell communication, conjugation, exogenous DNA acquisition, and competence (Ellison et al. 2018, Craig et al. 2019). Despite lacking a peptidoglycan cell wall, Archaea also exhibit a range of morphological forms similar to those of bacteria (Stetter 1999). A variety of selective pressures in the environment can influence cell morphology, although experimental evidence from complex prokaryotic communities in the field is still lacking. It is well established, however, that the peptidoglycan sacculus, a cross-linked polymer of glycan chains, dictates cell shape in most bacteria (Weidel et al. 1960, Liechti et al. 2014, Rast et al. 2017).

Analysis of prokaryotic shapes and morphological features are relevant to understand the adaptation strategies during low and high maintenance activities. Prokaryotic maintenance refers to all nongrowth components in a cell (van Bodegom 2007). Driving these maintenance activities requires energy expenditure for the regulation of internal pH, shifts in metabolic pathways, osmoregulation, cell motility, proofreading, establishing ion gradients, defence mechanisms, energy spilling, and macromolecular turnover (Russell 2007, van Bodegom 2007, Wikner and Vikström 2023). Existing knowledge of genes related to these maintenance processes and morphological features provide a theoretical basis to investigate their ecological significance in complex plankton communities. Few estimates of prokaryotic maintenance respiration in both field and mesocosm studies during different seasons indicate that it is higher during the winter as compared to summer conditions (Vikström and Wikner 2019, Verma et al. 2022). Also, a recent report from the Baltic Proper to Northeast Atlantic suggests that maintenance respiration constitutes a significant proportion of respiration in marine ecosystems (Wikner and Vikström 2023). The reciprocal and universal relationship between prokaryotic specific growth rate and growth efficiency established in metabolic theory, field, and experimental conditions postulate that the level of maintenance respiration can be manipulated with specific growth rate (Pirt 1982, Del Giorgio and Cole 1998, Verma et al. 2022).

We have not found studies in which the prokaryotic morphological features and the genes associated with maintenance activities were investigated in a complex prokaryotic community under controlled experimental conditions. The morphology and underlying genetics of prokaryotes have mainly been reported from pure cultures, and limited information exists about prokaryotic morphology in developed planktonic food webs. We believe that our work will provide a valuable extension to the already existing studies with the cultured isolates and to support the idea that basic principles seen with model species also apply to natural prokaryotic communities. This motivated us to analyse changes in prokaryotic morphology and differential abundance of genes in a mesocosm experiment to better understand factors regulating maintenance and growth-based activities. The fact that most maintenance activities are affiliated with basal metabolism made us hypothesize that common responses across taxa can also be detected in complex communities. Additionally, we propose that marine ecosystems with varying levels of maintenance respiration may exhibit noticeable effects on morphology and gene expression patterns. Based on these ideas, the following specific research questions were addressed: (1) Are there specific prokaryotic cell shapes and morphological features associated with maintenance and growth-based activities during winter and summer? (2) Are there specific genes that can explain the observed morphological features? (3) Is there a differential abundance of genes that indicate important maintenance activities?

## Materials and methods

### Experimental setup

The experiment was conducted in 12 mesocosm units under winter conditions at the Umea Marine Science Centre, Umea University, Sweden, located in the northwestern Bothnian Sea (63°34′ N, 19°15′ E). A full factorial experimental design was conducted with temperature (1°C and 10°C) and nutrient additions (no additions and addition of +59 µmol C dm^−3^) to control the specific growth rates of prokaryotes and maintenance respiration share in mesocosm treatments for 27 days, starting in March 2020. Four different sets of mesocosms in triplicate were used: C, control (1°C, no additions); N, (1°C, + nutrients); T, (10°C, no additions); and TN (10°C, + nutrients). The details of the experimental setup have been previously described (Verma et al. 2022). Our objective was to investigate the morphological and metabolic adaptations of prokaryotes under conditions of low and high maintenance respiration. To achieve this goal, we used the C and TN treatments which simulated winter (low productivity) and summer conditions (high productivity), respectively. The maintenance respiration share exhibits an exponential decrease with a corresponding increase in specific growth rates, with higher values observed in the C treatment and lower values in the TN treatment. Furthermore, we believe that the development of a well-established zooplankton community in the TN treatment by the end of the experiment, which was not detectable in the C treatment, further supports that the key characteristics of summer and winter conditions were achieved (Verma et al. 2022). The N and T treatments were not included in this analysis due to the lack of significant differences in the share of maintenance respiration (see Fig. 4, Verma et al. 2022). Furthermore, these treatments do not directly simulate other seasonal conditions but are included to ensure a full factorial design of the experiment. Specific data points from days 10 and 17 were selected due to significant differences in specific growth rates and maintenance respiration shares. For clarity in interpreting the results on a seasonal scale, we refer to the C treatment as winter conditions and the TN treatment as summer conditions in the discussion section.

### Prokaryotic variables

The estimations of prokaryotic abundance (PA), cell volume (BioVol), specific growth rate (µ), growth (PG), respiration (PR), specific respiration (*ρ*), and maintenance respiration (*ρ_m_*) were performed as previously described (Verma et al. 2022). Briefly, PA was estimated using direct epifluorescence microscopy (EpiM) (Zeiss Axioscope 5, Plan-Apochromat 63×/1.4, oil, ∞/0,17, Zeiss GmBH Germany) (Hobbie 1977). To eliminate the possibility of cell‒cell connections due to the greater abundance of cells, the formaldehyde-fixed samples were treated with Triton X‒100 [0.0001% (v/v)] followed by vortexing and sonication in an ice bath at 75 W for 30 s. Cell morphology and BioVol were estimated with image analysis software via neural network technology (Blackburn et al. 1998). PR was estimated by dark incubation of the samples in an incubator box with optodes as described previously (Wikner et al. 2013, Vikström and Wikner 2019). PG was estimated by tritiated thymidine incorporation (Fuhrman and Azam 1982, Smith and Azam 1992). The *ρ_m_* was calculated using the simplified Pirt model based on Model II linear regression with a major axis loss function (Vikström and Wikner 2019). The *ρ* and µ were calculated by dividing PR and PG, respectively, by PA.

### Scanning electron microscopy and energy dispersive X-ray analysis

The collection of water samples was carried out through a valve at a depth of 1.5 m into HCl (1 mol dm^−3^)-washed polycarbonate bottles, which were subsequently transferred to climate-controlled rooms at 1°C and 10°C for processing. To maintain the integrity of the microbial structures and avoid dehydration- and centrifugation-induced artefacts, we first employed negative staining (NS) for transmission electron microscopy (TEM). However, due to cell loss and analytical challenges, we utilized scanning electron microscopy (SEM) method with concentration steps to increase the cell density during processing. Initial test runs of prefiltered seawater samples (<1.2 µm, 5 µl) for SEM analysis resulted in a negligible number of cells after sample processing. Therefore, the cells were concentrated to achieve sufficient cell density on glass coverslips. Approximately 500–600 ml of prefiltered samples were concentrated on 0.2 µm, 47 mm polycarbonate track-etched filters (Sartorius) at −15 kPa. During filtration, the water samples were gently mixed with a pipette to resuspend and concentrate the cells 100-fold. The approximate time between the filtration and processing steps of electron microscopy was 15 min. The concentrated samples were then centrifuged at 10 000 × *g* for 10 min at 4°C, and the pellet was resuspended in 0.5 ml of fixative 1 [5% (v/v) glutaraldehyde in 0.2 M sodium cacodylate buffer] and incubated at 4°C for 10 min. After incubation, the samples were centrifuged again (10 000 × *g* for 10 min at 4°C), and the pellet was resuspended in 0.5 ml of fixative 2 [2.5% (v/v) glutaraldehyde in 0.1 M sodium cacodylate buffer] overnight at 4°C. Following incubation, another centrifugation step (10 000 × *g* for 10 min at 4°C) was performed, and the pellet was resuspended in 0.5 ml of fixative 3 (0.1 M sodium cacodylate buffer) before storage at 4°C until further processing. To prepare the samples for electron microscopy, glass coverslips (12 mm diameter, Ted Pella) were sterilized in 70% ethanol and coated with 10x diluted poly-l-lysine solution [0.01% (w/v), Sigma‒Aldrich] for 1 h, followed by air-drying on filter paper. The fixed samples were centrifuged for 5 min and plated onto poly-l-lysine-coated glass coverslips for 1 h. The coverslips with the samples were then transferred to a designated holder and dehydrated in a series of graded ethanol solutions ranging from 70% to 95% followed by absolute ethanol for 10 min. The samples were subjected to critical-point drying using a Leica EM CPD300 Critical Point Dryer. Prior to SEM analysis, the samples were sputter-coated with 5 nm of chromium (Cr) using a Quorum QT150-ES sputter coater. The morphology of the cells was analysed using a Carl Zeiss Merlin field-emission SEM at 5 kV and 150 pA. The different morphologies of cells were recorded as frequencies, i.e. number of times each feature falls into specific categories. The positions of the cells were also recorded during imaging and analysed using an energy-dispersive X-ray spectrometer (EDX; Oxford Instruments X-Max 80 mm^2^) at 7 kV and 250 pA for elemental composition. The elemental composition within a cell was acquired through point analysis at two-three different positions, including the background control, and the average value was calculated relative to Cr. The elemental composition of different elements such as carbon (C), nitrogen (N), phosphorus (P), oxygen (O), sodium (Na), aluminium (Al), silicon (Si), potassium (K), titanium (Ti), and zinc (Zn) were recorded.

### Extraction of membrane vesicles

Despite the diverse roles of membrane vesicles in the marine ecosystems, their regulation and abundance estimates are still lacking. Few estimates of their abundance are known from both high nutrient and oligotrophic marine ecosystems (Biller et al. 2014). However, data from oligotrophic brackish seawater communities are currently lacking, motivating their quantification in this study. Due to the large volume of water required for membrane vesicle extraction, this extraction was performed at the end of the experiment rather than on days 10 and 17, as for other variables. A final volume of 24 l each from the C and TN treatments was collected (8 l each from triplicate mesocosm treatments). The samples were first prefiltered through a 90-µm mesh filter to remove meso-zooplankton and other particulate matter, followed by ultracentrifugation using a 300-kDa Ultrasette tangential flow filter (Pall, Port Washington, NY, USA). The amounts of retentate samples collected were 700 and 550 ml for the C and TN treatments, respectively, and the samples were preserved at −80°C until the extraction of vesicles. The samples were thawed and sterilized by filtration through a 0.22-µm polyvinylidene difluoride membrane filter (Millipore, Merck Chemicals and Life Science, Solna, Sweden). The filtrate was then subjected to ultracentrifugation at 100 000 × *g* for 3 h at 4°C using a 45-Ti rotor (Beckman Instrument). Since no clear pellet was visible at the bottom of the centrifuge tube, we skipped purifying the vesicle fraction by density gradient centrifugation. The supernatant was discarded, and the pellet was mixed with 500 µl of Tris–HCl buffer (pH 7.5) and stored at −20°C. We referred to this fraction of the extracted vesicles as ‘particles’, which included both vesicles and phages. Protein content was estimated using a bicinchoninic acid protein assay kit (Thermo Scientific Pierce^TM^, Rockford, IL, USA) according to the manufacturer’s instructions. The protein samples were precipitated with 50% (w/v) trichloroacetic acid at a 1:5 ratio, incubated on ice for 30 min, and then centrifuged at 12 000 × *g* for 15 min at 4°C. The supernatant was discarded, and the pellet was dissolved in 10 µl of 20 mM Tris–HCl buffer (pH 7.5). The protein samples were then subjected to 13.5% SDS‒PAGE with a discontinuous buffer system and run at a constant voltage of 120 V for 2‒2.5 h, followed by silver and Coomassie brilliant blue staining following standard procedures.

### Nanoparticle tracking analysis

Nanoparticle tracking analysis (NTA) was performed to determine the size distribution and number of particles (including both vesicles and phages) using a NanoSight NS300 instrument (Malvern Instruments, UK) equipped with NTA 3.1 software. For all recordings, a camera level of 12 and a detection threshold of 4 were applied. The samples were diluted 1:100 in double distilled water (ddH_2_O) to achieve a particle concentration ranging from 10^10^ to 10^11^ particles ml^−1^. Five 60 s videos were analysed for each sample to calculate the average abundance of particles with respect to the corresponding diameter.

### TEM

NS for TEM was performed to confirm the identity of membrane vesicles in the extracted particles. Five microlitres of extracted particles was placed on Formvar and carbon-coated copper grids and 1.5% uranyl acetate was used for NS before observation via TEM (Thermo Fischer Scientific Talos L 120C). The microscope was operated at an accelerating voltage of 120 kV, and the data were collected with a Ceta 16 M detector using Velox software.

### Metatranscriptomic analysis

Seawater was first prefiltered through 3-µm filters and collected on 0.22-µm Sterivex filters to conform with current standards as mentioned earlier (Pontiller et al. 2022, Massing et al. 2023). The prefiltration was done to specifically remove particle associated prokaryotic cells and to minimize the risk of clogging 0.22-µm Sterivex filters. Then, 1.8 ml of RNAlater was added to 0.22-µm Sterivex filters, and the samples were stored at −80°C until extraction. Total RNA was extracted using a protocol adapted from Poretsky et al. (2009) and an RNeasy Mini kit (Qiagen). The 0.22-µm Sterivex filters were thawed on ice, and the RNAlater reagent was removed before the filters were cut with a razor. Filters were placed in tubes containing a 1-ml solution consisting of RLT-lysis buffer, β-mercaptoethanol (10 µl ml^−1^ RLT buffer) and 2 spoon 200 µm zirconium beads (OPS diagnostics). The cells were lysed by vortexing for 15 min, followed by centrifugation at 4°C for 5 min at 5000 × *g*. The supernatants were transferred to tubes containing 1 volume of 70% ethanol and mixed by repeated pipetting. Extraction was continued using the RNeasy Mini Kit following the manufacturer’s instructions. Total RNA was eluted twice using 30 µl preheated RNase-free water. Samples were treated with DNase using a TURBO DNA-free kit (Thermo Fisher Scientific) following the manufacturer’s protocol. The RNA yield was measured using a Qubit and Nanodrop. Before transcriptomic analysis, each sample was checked for genomic contamination using 16S ribosomal RNA (rRNA) gene polymerase chain reaction (PCR) and gel electrophoresis. rRNA was depleted using a RiboMinus transcriptome isolation kit and RiboMinus Concentration Module (Thermo Fisher Scientific) following the manufacturer’s protocol. Ribosomal RNA molecules were then removed by binding to magnetic beads, and only the rRNA-depleted fraction was further concentrated and purified using silica spin columns. RNA was amplified using the MessageAmp II-Bacteria RNA Amplification Kit (Thermo Fisher Scientific) following the manufacturer’s instructions. Finally, the final RNA was quantified and stored at −80°C until sequencing. Samples were sequenced on the NovaSeq 6000 (Illumina, Inc.) platform with 2 × 150 bp size. The samples were processed from the nf-core using the nf-core metatdenovo assembly pipeline (prerelease version 1.0.1) (Ewels et al. 2020, Di Leo et al., in preparation). Briefly, samples were subjected to quality control and trimmed using Trimgalore (version 0.6.6, https://github.com/FelixKrueger/TrimGalore) as a wrapper for fastQC (version 0.11.9; Andrews 2010) and multiQC (version 1.8; Ewels et al. 2020), followed by primer removal using Cutadapt (version 3.2; Martin 2011). The sequences were assembled using megahit (version 1.2.9; Li et al. 2015), and the open reading frames were identified with prodigal (version 2.6.3; Hyatt et al. 2010) and functionally annotated with EGGNOG-mapper (version 2.0.8–2; Huerta-Cepas et al. 2017). Subsequently, the open reading frames were supplemented with e-values and bitscores using kofamscan (version 1.3.0) to improve annotation (Aramaki et al. 2020). Taxonomy was assigned with EUKulele (version 2.0.5, Krinos et al. 2020) against the genome taxonomy database (GTDB, version R207, Parks et al. 2018), outside of the nf-core pipeline. Sequences were then imported into R (version 3.6.3, R core team), where nonprokaryotic reads were manually removed, and functionally annotated genes were filtered based on an e-value threshold of 10^−5^. The best hit for each KEGG orthology term from the Kyoto Encyclopedia of Genes and Genomes (KEGG) based on e-values and bitscores was then selected. KEGG pathway analysis was performed on the prokaryotic fraction of the larger KEGG categories spanning multiple pathways.

### Statistical and bioinformatic analysis

Triplicate mesocosm units of the C and TN treatments were sampled at days 10 and 17. Based on the data distribution, both parametric and nonparametric tests were employed depending on the Shapiro‒Wilk test for normally distributed data. Paired *t*-tests were used to compare differences between treatments for all prokaryotic variables. Prokaryotic cell shapes were compared using a paired *t*-test in EpiM and the Mann‒Whitney U test in SEM. The Pearson’s chi-square test (*χ*^2^ test) was used to examine a potential association between two categorical values (i.e. treatments and the morphological features). Fisher’s exact test was used for the morphological features of EPS and cell division, as the expected frequency of features was less than five. The Mann‒Whitney U test was used to compare the elemental composition among treatments. Statistical analysis of the metatranscriptomic data was performed using generalized linear models from the EdgeR package in R (Chen et al. 2016). The DE or abundant genes were screened to identify genes involved in relevant processes.

## Results

### Contrasting specific growth rates and maintenance respiration modes

To investigate the impact of simulated seasonal conditions, different prokaryotic variables were estimated for the C (simulating winter conditions) and TN (simulating summer conditions) treatments (Supplementary Figure S1). The specific growth rate (µ) was significantly greater in the TN treatment than in the C treatment, while the maintenance respiration share (*ρ_m_*/*ρ*) was significantly greater in the C treatment than in the TN treatment (paired *t*-test, *n* = 6, *P* < .05; Supplementary Figure S1). Except for *ρ_m_*/*ρ*, most of the other prokaryotic variables (PR, PG, µ, and BioVol) were significantly greater in the TN treatment than in the C treatment (paired *t*-test, *n* = 6 each, *P* < .05), except for PA and *ρ*, which showed no significant differences between treatments. The descriptive statistics of these variables are presented in Supplementary Table S1.

### Simulated seasonal effects on cell shape

**Figure 1.**
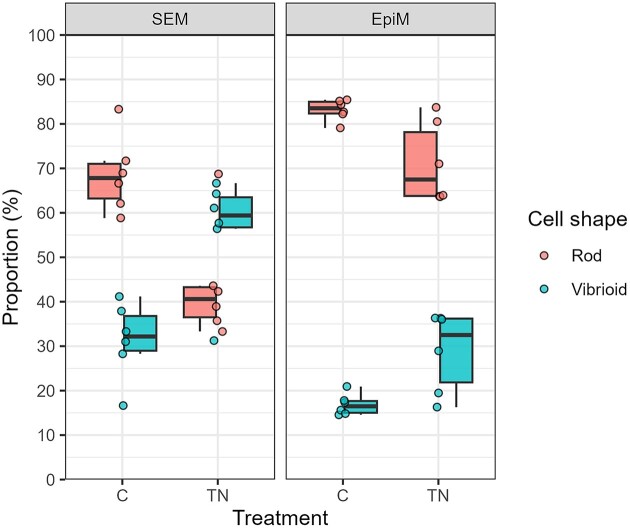
Cell shape proportion of prokaryotes in the C (simulating winter conditions) and TN (simulating summer conditions) treatments analysed by SEM and EpiM. The box and whisker plot shows the median as a horizontal line in the box and the interquartile range (IQR or middle 50% of the dataset), with whiskers extending to the minimum and maximum values from the box edges (1.5 times the IQR). Each data point shows the average distribution from the triplicate measurements. See Supplementary Table S2 for more details.

Both EpiM and scanning electron microscopy (SEM) revealed that rods and vibrioids were the predominant cell shapes in both the C and TN treatments (Fig. 1). SEM analysis demonstrated a clear shift in cell shape from rods dominating the C treatment to vibrioids dominating the TN treatment. This shift towards vibrioids in the TN treatment was not detected by EpiM, possibly due to the lower optical resolution of EpiM. The estimates of prokaryotic cells from EpiM were significantly greater than those from SEM (35 × greater for C and 28 × greater for TN; Fig. 1). However, SEM, with its markedly higher resolution (1200–1500 times higher than that of EpiM), more accurately determines the cell shape. In addition to rod and vibrioid shapes, spiral and coccoid shapes were identified by SEM, occurring at a proportion of 1% each for spiral and coccoid shapes in the C treatments and 6.3% for coccoid shapes in the TN treatments (Supplementary Figure S2). The abundance of rods was significantly greater in the C treatment than in the TN treatment, as confirmed by both the EpiM (Fig. 1, Supplementary Table S2; paired *t*-test, *n* = 6, *P* < .05) and SEM (Fig. 1, Supplementary Table S2; Mann‒Whitney U test, *n* = 6, *P* < .05) results. On the other hand, the abundance of vibrioid cells was significantly greater in the TN treatment than in the C treatment according to both microscopy methods (Fig. 1, Supplementary Table S2; Mann‒Whitney U test, *n* = 6 each, *P* < .05).

### Simulated seasonal effects on morphological features

**Figure 2.**
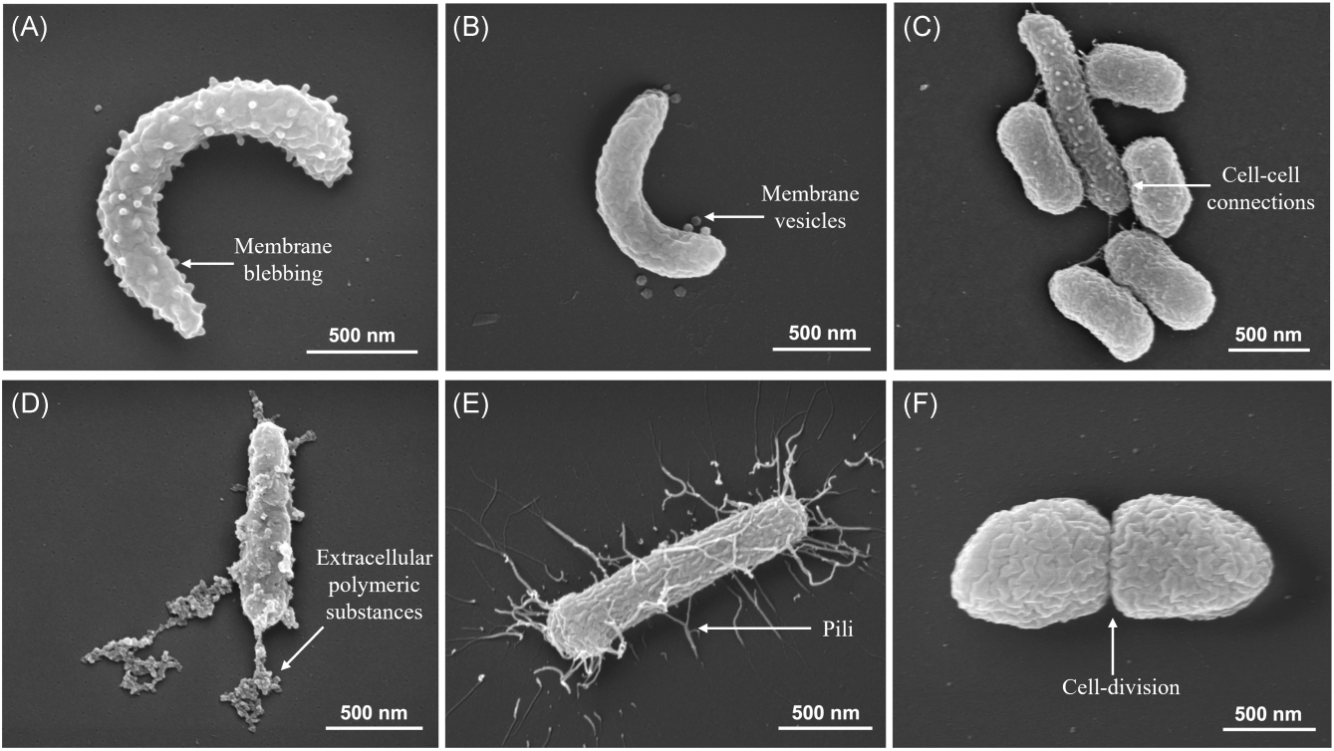
SEM revealed different categories of prokaryotic morphological features in the C and TN treatments. Arrows point towards the specific morphological features in each panel. (A) membrane blebbing, (B) membrane vesicles, (C) cell‒cell connections, (D) EPS, (E) pili, and (F) cell division. Scale bar, 500 nm.

**Figure 3.**
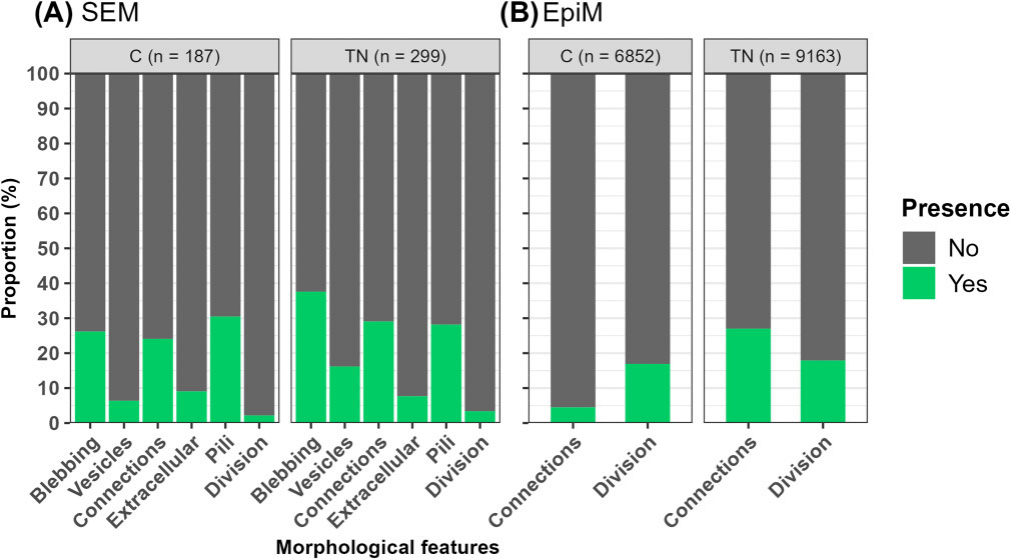
Morphological features of prokaryotes analysed using (A) SEM and (B) EpiM. The *n* values alongside the treatment panel refer to the number of cells analysed. The *x*-axis shows different classes of morphological features (full names omitted for clarity), and the *y*-axis shows the proportion of the presence and absence of morphological features. Figure 2 shows a description of these morphological features.

Based on the high resolution of the SEM image (80–100 k×), six distinctive morphological features were identified in both the C and TN treatments. These were membrane blebbing, cell‒cell connections, cell division, EPS, and the presence of pili and membrane vesicles (Figs 2 and 3). Further representative images of these morphological features are shown in Supplementary Figure S3. To ensure that our processing method did not affect the presence of flagella, we analysed the marine bacterium *Marinomonas* sp. GOBB3-320, which was isolated from the Baltic Sea, using the same SEM method. The clear presence of flagella in this isolate indicated that our SEM processing method did not influence its occurrence (Supplementary Figure S4).

Most of the prokaryotes did not show any specific morphological features in either the C (69.6%–97.8%) or TN (62.4%–96.9%) treatment (Fig. 3A). All the morphological features were more abundant in the TN treatment, except for the presence of pili, for which there were no significant differences (C: 30.5%; TN: 28.2%, *χ*^2^ test, *P* > .05; Fig. 3A). Interestingly, analysis of a specific cell shape and different cell shapes in combination resulted in different statistical outcomes (Table 1). Analysis of all cell shapes (rod and vibrioid) revealed a significantly greater occurrence of membrane blebbing (C: 26.2%; TN: 37.6%) and membrane vesicles (C: 6.4%; TN: 16.2%) in the TN treatment (Fig. 3A). These morphological features also exhibited significant associations and higher odds ratios in the TN treatment group (Fig. 3A, Table 1; *χ*^2^ test, *P* < .05). Among the vibrioid-shaped cells, TN treatment had a significantly greater effect on cell‒cell connections (C: 18%; TN: 32.8%) and membrane blebbing (C: 32.7%; TN: 55.2%) (data not shown). Similarly, these morphological features also showed a significant association with a greater odds ratio in the TN treatment (Fig. 3A, Table 1; *χ*^2^ test, *P* ≤ .05). Rod cells, on the other hand, did not show any specific morphological features (Table 1). Among the six different morphological features and a total of 57 different combinations, only a few significant combinations were observed in the TN treatment (Supplementary Table S3). Notably, cell‒cell connections were a common morphological feature in all cases. Rod-shaped cells with a combination of cell‒cell connections, the release of EPS and the presence of pili were significantly associated with a greater odds ratio in the TN treatment (*χ*^2^ test, *P* = .05; Supplementary Table S3). The analysis of morphological feature combinations for individual vibrioid cell shapes and all cell shapes together revealed similar significant associations of cell‒cell connections with both pili and membrane blebbing, with a greater odds ratio in the TN treatment than in the C treatment (*χ*^2^ test, *P* ≤ .05; Supplementary Table S3). Similar to the SEM results, the majority of the cells in the EpiM also lacked morphological features in the both treatments (C: 83%–95%; TN: 73%–82%; Fig. 3B). Despite low resolution in EpiM (63 ×), two morphological features, cell‒cell connections (C: 4.5%; TN: 27%) and cell division (C: 17%; TN: 18%), were identified (Fig. 3B). However, it was not possible to identify cell-specific morphological features as in the SEM analysis. The only significant feature of the combination of rods and vibrioids was cell‒cell connections in the TN treatment group, with a high odds ratio (*χ*^2^ test, *P* < .001; Table 1).

**Table 1.** Chi-square test (*χ*^2^) of the difference in morphological features between the C and TN treatments. The odds ratio (TN/C) represents the odds of having a specific morphological feature during summer compared to winter.

Microscopy (shape)	Morphological features	*χ* ^2^-test, *P*-value	Odds ratio (TN/C)
EpiM (rods and vibrioids)	Cell‒cell connections	238.1, < .001	6.0
SEM (rods and vibrioids)	Membrane blebbing	4.4, .03	1.9
	Membrane vesicles	7.5, .006	2.8
SEM (vibrioids)	Cell‒cell connections	3.6, .05	2.2
	Membrane blebbing	6.5, .01	2.5
SEM (rods)	ND	–	–

ND = not detected. – = No data.

SEM–EDX analysis revealed that the ratios of the relative atomic % of major cell elements (C:N:P) to phosphorus in the C and TN treatments were 85:10:1 and 63:7:1, respectively (Supplementary Table S1, Supplementary Figure S5). The ratios of C:P and N:P did not significantly differ between prokaryotes in the C and TN treatments (Mann‒Whitney U test, *n* = 127 each, *P* > .05). In addition to C, N, and P, other elements, such as O, Na, Al, Si, K, Ti, and Zn were also detected in the samples (Supplementary Figures S6A and B). However, these elements were not included in the analysis due to their potential source from glass slides, fixatives, buffers, and the possibility of overlap with elements originating from the samples.

### Vesicle and phage abundances

The protein concentration in the particles, assumed to be vesicles and phages, was 4.1 times greater in the TN treatment than in the C treatment (Supplementary Table S1, Supplementary Figure S7). Silver staining, applied to the already stained Coomassie gel, enhanced the banding patterns due to its increased sensitivity (Supplementary Figure S7A and B). This banding pattern with a low protein concentration of particles in the C treatment group was also observed via TEM, which revealed a lower occurrence of membrane vesicles (Supplementary Figure S8A–C). The higher protein concentration of the particles in the TN treatment group was supported by the greater occurrence of membrane vesicles and phages in addition to some filaments (Supplementary Figure S8D–F). An interaction between membrane vesicles and a phage was also observed in the TN treatment (Supplementary Figure S8G and H). The abundance of particles was significantly greater in the TN treatment than in the C treatment (Supplementary Figure S9A, Wilcoxon signed-rank test, *n* = 26, *P* < .01), whereas the abundance of prokaryotes did not differ (Wilcoxon signed-rank test, *n* = 3, *P* > .05; Supplementary Figure S9B). The abundance of particles was 50 times and 79 times greater than that of prokaryotic cells in the C and TN treatments, respectively, with no significant differences between the treatments (Supplementary Figure S9C).

### Expression of genes related to maintenance activities and morphological features

**Figure 4.**
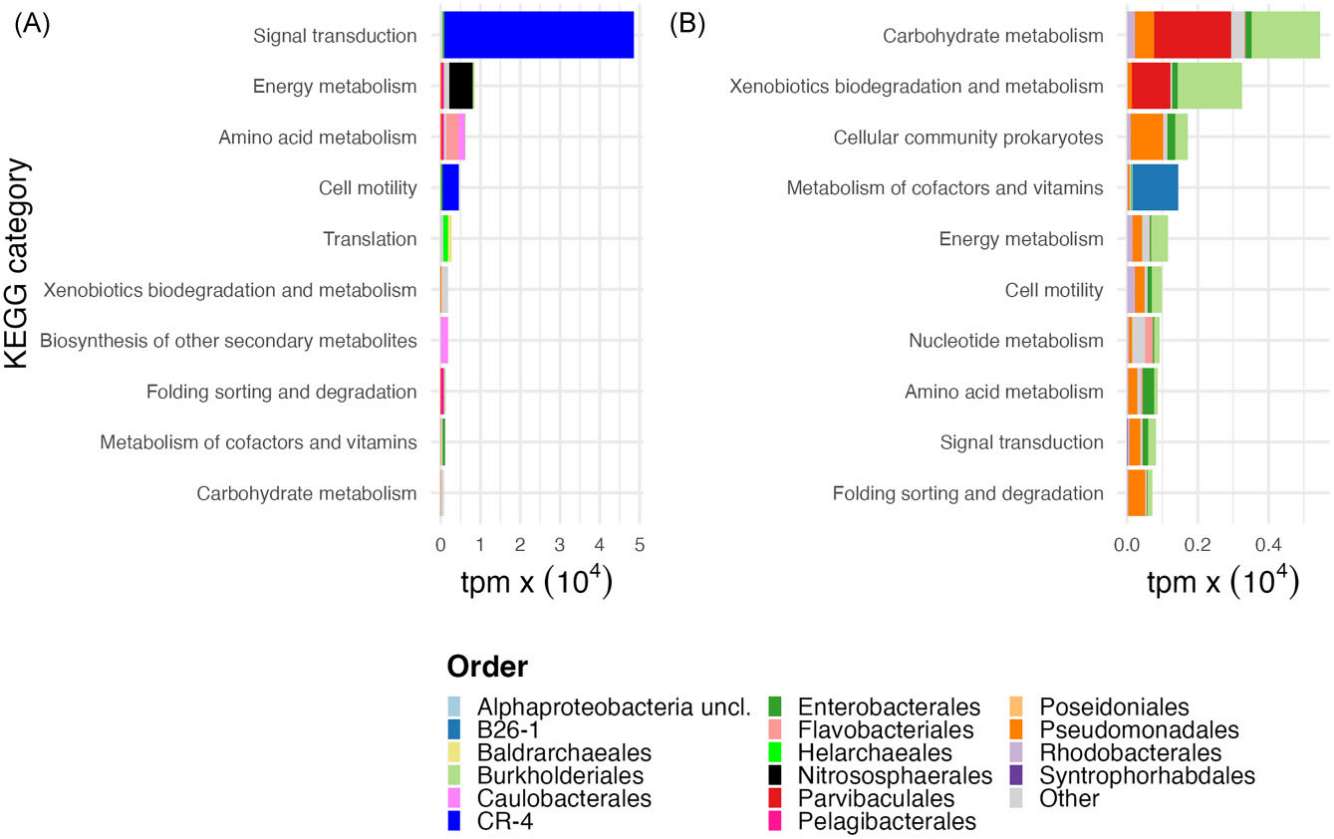
Differentially abundant KEGG categories as a function of tpm in the C (A) and TN treatment (B). Different colour shadings in the barplot represent taxonomy at the order level.

The sequencing statistics for the C and TN treatments are presented in Supplementary Table S4. Out of a total of 5498 gene functions identified in the investigated metatranscriptomes, EdgeR analysis identified 79 and 242 gene functions that were expressed at a significantly greater relative abundance in the C and TN treatments, respectively (Supplementary Table S5A). Collectively, these gene functions accounted for 32 809 and 26 821 average transcripts per million (tpm) in the C and TN treatments, respectively. The differentially expressed (DE) and non-differentially expressed (non-DE) genes were summarized over KEGG database, categories to ascertain broad metabolic affiliations (Kanehisa and Goto 2000; Supplementary Table S5A and B). A summary of the 12 most differentially abundant KEGG categories based on tpm values and taxonomic identity at the phyla and order level revealed significant differences at the levels of categories and transcripts in both the C and TN treatments (Fig. 4, Supplementary Figure S10). Both treatments showed eight similar categories, with few categories specific to C (translation, biosynthesis of secondary metabolites) and TN treatment (cellular community prokaryotes, nucleotide metabolism) (Fig. 4).

At the level of KEGG categories, signal transduction was differentially abundant in the C treatment followed by energy metabolism and other categories. At the order level, CR-4 and Nitrososphaerales were the dominant members of the archaea, along with bacterial members of Flavobacteriales and Caulobacterales (Fig. 4). At the phyla level, the differentially abundant KEGG categories belonged to Asgardarchaeota and Thermoproteota with some members of Proteobacteria (Pseudomonodota as per latest GTDB taxonomy) and Bacteroidota (Supplementary Figure S10). In the TN treatment, the abundant KEGG categories belonged to carbohydrate metabolism and xenobiotics biodegradation and metabolism (Fig. 4). At the order level, Parvibaculalaes, Burkhoderiales, and Pseudomonadales were the dominant taxa (Fig. 4). It is important to note the level of differential expression of the first abundant category i.e. ‘Signal transduction’ was approximately >10 times higher in the C treatment as compared to ‘Carbohydrate metabolism’ in TN treatment (Fig. 4).

**Figure 5.**
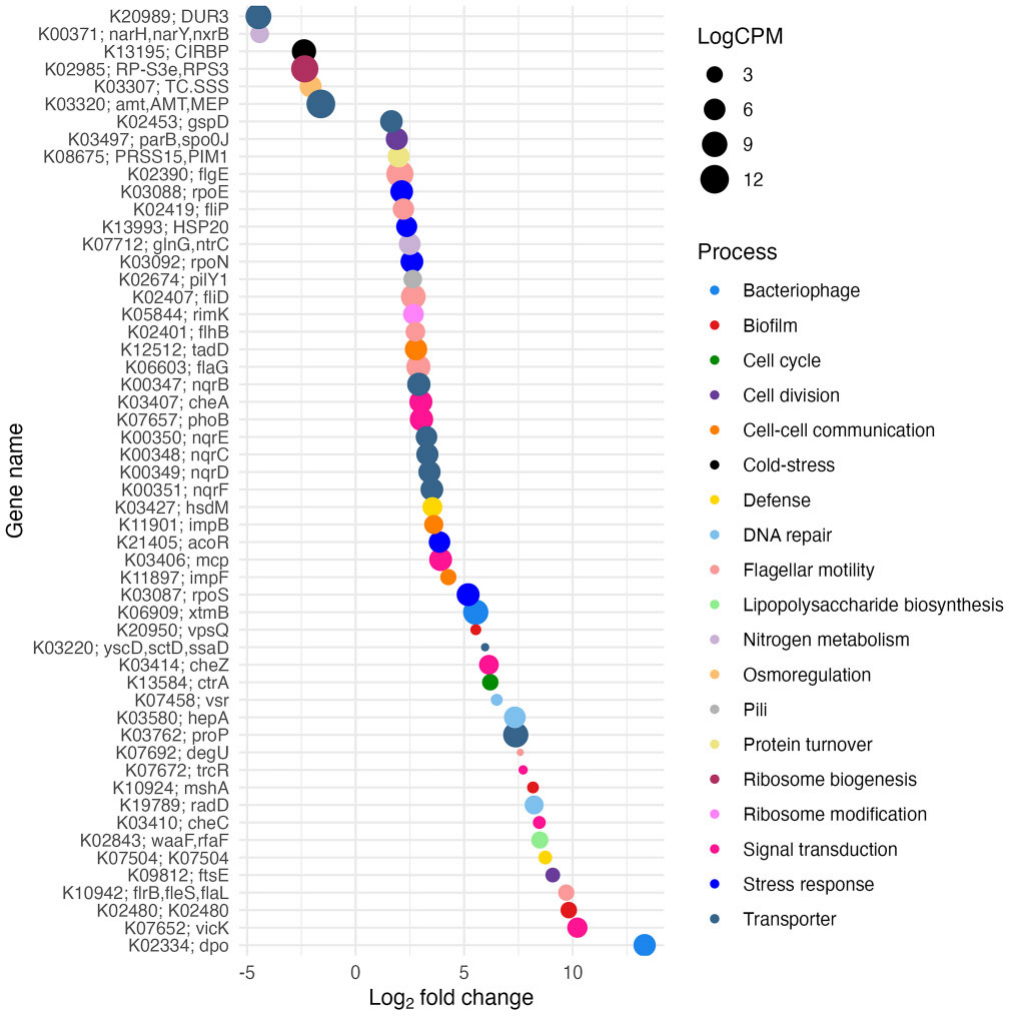
Differential abundance of genes associated with morphology and maintenance activities in the C and TN treatments based on KEGG database. The *x*-axis shows the log2-fold change values (logFC) of gene functions between treatments, and the *y*-axis shows the gene functions involved in relevant processes, scored by logFC* average log2-counts per million (logCPM). Negative logFC values indicate higher gene expression in the C treatment, and positive values indicate higher gene expression in the TN treatment. The legend indicates the metabolic process to which genes are represented by different colours. The extent of gene expression is shown as log2-counts per million transcripts (logCPM).

Among the functions of the DE gene, 54 KEGG categories with gene functions related to morphological features and maintenance activities were associated with the C and TN treatments (Supplementary Table S5C, Fig. 5). In the C treatment, genes related to transporters (*n* = 4), nitrogen metabolism (*n* = 3), ribosome biogenesis (*n* = 2), osmoregulation, and cold stress (*n* = 1 each), were differentially abundant (Fig. 5). In the TN treatment, eight relevant KEGG categories with gene functions related to transporters (*n* = 8), flagellar motility (*n* = 7), defence (*n* = 4), biofilm formation, DNA repair (*n* = 3 each), cell division (*n* = 2), vesicle biogenesis, cell–cell communication, pili, protein turnover, and ribosome modification (*n* = 1 each) were differentially abundant (Fig. 5). Among the a priori list of genes of interest related to morphology and maintenance (Supplementary Table S6), only one gene in the C treatment group and four genes in the TN treatment group were identified (Supplementary Table S7). The identified gene in the C treatment was part of the signal transduction KEGG category. In the TN treatment, genes related to environmental stress, defence mechanism, degradation of aromatic compounds, and LPS biosynthesis needed for vesicle biogenesis were identified (*n* = 1 each) (Supplementary Table S7).

## Discussion

### Cell shape adaptation to seasonal conditions

Prokaryotes display a wide range of cell shapes that enhance their fitness in diverse environments (Jiang et al. 2015, Yang et al. 2016, Orr et al. 2020). Under winter conditions, rod-shaped cells were more common (Fig. 1, Supplementary Table S2), possibly because this shape offers a selective advantage for efficient substrate uptake, owing to its high surface-to-volume ratio (Beveridge 1988). This surface-to-volume ratio of rod cells remains constant even during their growth (Tamames et al. 2001), potentially conferring a selective advantage at a low specific growth rate and a high maintenance cost (i.e. respiration) during winter. Conversely, under summer conditions, the increase in both cell numbers (Supplementary Figure S1A) and vibrioid cell shapes (Fig. 1, Supplementary Table S2) can be attributed to the favourable environment for prokaryotic growth facilitated by higher temperatures and increased nutrient availability. This finding aligns with earlier research showing that vibrioids are more abundant in high cell density populations than in low cell density populations (Henrici 1928, Bartlett et al. 2017). Vibrioid shapes with curvature also have a selective advantage in facilitating movement through viscous fluid due to their curved morphology compared to rod cells with no curvature (Seuront et al. 2010, Prairie et al. 2012). The movement of any organism in seawater is influenced by viscosity, which decreases with increasing temperature (Taucher et al. 2014). However, a positive correlation between seawater viscosity and temperature due to mucus exopolymers secreted from phytoplankton and prokaryotes has also been reported (Jenkinson and Biddanda 1995, Seuront et al. 2010). We therefore assume that the seawater viscosity is greater under summer conditions due to zooplankton excretion, prokaryotic abuandance, viral cell lysis, exopolymers, proteins, and macromolecules. This was further supported by the zooplankton biomass, which was 10× greater under summer conditions than under winter conditions at the end of the mesocosm experiment (data not shown), plausibly elevating the organic substrate supply during days 10 and 17 of the experiment. Also, the 4× greater protein concentration of particles during the summer conditions supported higher seawater viscosity than in the winter conditions (Supplementary Table S1).

Taken together, these findings indicate that transition from winter to summer shifts the dominance of rod-shaped cells to vibrioids, which together dominated the morphological types. One could argue about the potential outcomes if the experiment had started in summer and transitioned to winter conditions. Although it would be possible to control the temperature, removing the extensive remineralization of cellular components by different zooplankton communities remains impractical. However, we cannot exclude the possibility of different results and outcomes if we had started with different starting communities. Conducting additional experimental setups addressing similar questions in various marine ecosystems would be valuable for gaining further insights.

### Morphological features: role and significance

Due to the higher resolution provided by the SEM method, we were able to analyse a variety of prokaryotic morphological features (Fig. 3). Nonetheless, analysing many cells via SEM posed significant challenges, which required considerable microscopy time and effort. Additionally, cell loss during processing steps may have influenced the types of cells available for analysis. To address some of the biases inherent in this method, we scanned several different fields in a zig-zag pattern to capture representative images of cells on glass coverslips and ensured an adequate number of prokaryotic cells were imaged (Supplementary Table S2). However, we cannot exclude the possibility that the SEM method may affect the morphotype proportion. No significant morphological features were reported under low productive winter conditions, as cells may not find it advantageous to allocate energy towards developing such features unless they directly enhance overall fitness. In contrary, among the six different morphological features examined (Fig. 2), membrane blebbing, membrane vesicles, and cell–cell connections demonstrated a significant association with prokaryotic communities under summer conditions.

The greater proportion of membrane blebbing in both the winter and summer seasons (>25%), with a notably greater occurrence in summer, suggests some degree of seasonal regulation of this feature. The significantly higher distribution of membrane vesicles resulted from the regulated release through budding or blebbing of the outer or inner membrane. Given the abundance of this morphological feature, further investigations of their specific roles in marine ecosystems are needed. A previous electron microscopy-based study of cold-adapted Antarctic strains revealed a decrease in vesicle concentration with increasing temperature (Frias et al. 2010); however, the opposite effect of increased vesicle production during summer conditions (2.5 times greater) was observed in our study (Fig. 3A). This could be due to the presence of diverse prokaryotic communities and their complex interplay with temperature and nutrients, which can alter the release of membrane vesicles in natural ecosystems. However, literature analysis also revealed that other factors, such as growth conditions, stress, and membrane structure, can affect the release of membrane vesicles in the environment (MacDonald and Kuehn 2012, Zlatkov et al. 2021). Biller et al. (2014) provides an estimate of the pure vesicle fraction from the Sargasso Sea and coastal surface waters (10^5^–10^6^ ml^−1^); however, its comparison with our data was not relevant due to presence of both phages and vesicles in our study (Supplementary Figure S9A). Based on NTA and TEM, it was clear that the abundance of particles (vesicles and phages) was greater under summer conditions than under winter conditions (Supplementary Figure S9). An interesting role of membrane vesicles as a defence barrier to protect prokaryotes from phage was observed in our extracted vesicles under summer conditions (Supplementary Figure S8G and H), which is consistent with the defence role of vesicles described in previous reports (Manning and Kuehn 2011, Biller et al. 2014). It is thus clear that prokaryotes allocate their resources to the production of membrane vesicles due to their broad functional roles, and their production can vary due to seasonal conditions.

The cell‒cell connections primarily occurred during the summer conditions, which agrees with the findings of Kent et al. (2018), where increased biofilm formation is seen as an adaptation mechanism to increased temperature. The cell‒cell connections observed during the summer in our analysis were unlikely due to random aggregation of cells during sample processing. This conclusion was supported by a calculation indicating that ~98.3%–99% of the 0.2-µm filter (∅ 25 mm) remained free of cells. This was based on the filtration of 5 ml seawater, with a prokaryotic concentration ranging from 0.5–5 × 10^6^ cells ml^−1^ (Supplementary Figure S1A), and assuming an average cell size of 0.6 µm. Therefore, the presence of cell‒cell connections likely reflected genuine morphological interaction influenced by treatment effects rather than methodological artefacts. Notably, cell‒cell connections can be induced either by the presence of EPS (Decho and Gutierrez 2017) or by the presence of extracellular appendages such as pili (Singhi and Srivastava 2020). The greater predominance of cell‒cell connections during both winter and summer (>20%) indicated that this feature is a preferred mode of communication among cells irrespective of cell shape and seasonal conditions. Both EPS-like structures and pili were present in our analysis (Fig. 2D and E) and likely responsible for cell‒cell connections as observed in combinations with different morphological features (Supplementary Table S3). Prokaryotic EPS constitute a major component of the total dissolved organic carbon pool in the ocean (Aluwihare et al. 1997). The distribution of EPS (<10%) in both winter and summer indicated that only a small fraction of the prokaryotic communities allocated their resources to EPS secretion. The similar occurrence of pili in both the winter (32%) and summer treatments (29.5%) (Fig. 3) suggested that, irrespective of differences in temperature and nutrient availability, the presence of pili was an important morphological feature for various metabolic functions (see the section ‘Introduction’). The similar frequency of dividing cells (FDC) in winter and summer conditions was somewhat unexpected, given the higher specific growth rate of prokaryotes in summer conditions (Fig. 3). The FDC was reported to be low in SEM (<5%) and high in EpiM (15%–20%), as EpiM provided higher estimates of FDC in the natural environment due to better coverage of the prokaryotic community (Hagström et al. 1979). Although the differences in these morphological features between the winter and summer conditions were not discernible even for EpiM, where we had better coverage, it remains possible that the frequency (mentioned as proportion here) of cell division in specific prokaryotic taxa might be similar within a broad range of temperatures and nutrient availability. However, validation of this statement requires further investigation with natural prokaryotic communities and cultured isolates in an experimental setup.

All the morphological features mentioned above could provide a range of functions and can improve the overall fitness of prokaryotes by supporting cell communication, nutrient acquisition, attachment, information flow, and genetic exchange. Thus, investment by the cells in these morphological features during the summer season can be explained by a greater energy supply through solar irradiation; thus, higher temperatures and more bioavailable nutrients promote improved growth conditions for prokaryotes. Interestingly, no cells with flagella were observed in our electron micrographs under winter or summer conditions. This is in accordance with previous reports suggesting that cells with flagella are rare in energy- and nutrient-limited environments (Lever et al. 2015, Kempes et al. 2017, Ferreira et al. 2019), as exemplified by the abundant groups of marine bacteria, SAR 11 and *Prochlorococcus*, which are nonmotile in nature (Giovannoni et al. 2005). The absence of flagella in addition to the lack of significant morphological features during winter could be a result of their adaptation to oligotrophic and cold sub-Arctic environments, with trade-offs in resource allocation and potential benefits in terms of energy conservation and survival strategies. This absence aligns with the broader ecological understanding of how organisms respond to energy and nutrient limitations.

In addition to the effects of growth rate and temperature on prokaryotic morphology, grazing by bacterivores also influence cell size and morphology. Already early aquatic microbial ecology studies showed that grazing by different bacterivores is size-selective. For instance, Andersson et al. (1986) showed that bacterivores, such as flagellates, reduce the median cell volume of pelagic bacteria by 47% through size-selective grazing on cells larger than 0.2 µm^3^. Similarly, Gonzalez et al. (1990) showed that the small average size of *in situ* bacterioplankton in estuaries result from size-selective grazing by flagellates and ciliates, as evidenced by the varying rates at which these bacterivores consume fluorescently labelled bacteria. Beyond size-selective grazing, significant morphological changes in bacteria have also been observed in oligotrophic coastal systems and sea water cultures, including formation of grazing-resistant elongated filamentous cells and shifts in community composition (Simek et al. 1997, Weinbauer et al. 2019). However, we have not found high-resolution studies of the effects of bacterivore grazing on prokaryotic morphology by electron microscopy, a question which could be addressed in future research.

It is pertinent to mention that the prokaryotic morphologies analysed in our study were first prefiltered through <1.2 µm pore sizes, selecting the free-living prokaryotes and excluding the particle-attached cells. The morphological strategy of particle-thriving prokaryotes can be different from that of free-living prokaryotes. Future advancements in microbial ecology with a particular focus on transmission and cryo-electron microscopy are thus needed to enable *in situ* analysis of cell morphology and their complex interactions with other organisms of food webs that are free from processing biases and artefacts.

### Linking morphological features to genes during summer conditions

The morphological features related to cell‒cell connections, pili and membrane vesicles were supported by the genes observed under summer conditions (Fig. 5). Many of the genes identified in our analysis supported cell‒cell communication through their role in cell aggregation through biofilm formation (Fig. 5). The gene *mshA* plays an important role in the production of type IV mannose-sensitive hemagglutinin pilus required for cell adhesion and biofilm formation (Floyd et al. 2020). Another gene, *vpsQ* facilitates the synthesis of *Vibrio* polysaccharide crucial for biofilm formation and enhancing cell–cell communication (Fong et al. 2010). The tight adherence protein *tadD*, identified in our analysis, is part of the Tad secretion system, which secretes surface pili to support cell adherence and biofilm formation (Kachlany et al. 2000). A recent report by Kent et al. (2018) reinforced our findings regarding differential expression of biofilm formation during summer conditions. The study by Kent et al. (2018) highlights genetic alterations in strains of the *Roseobacter* clade, with genes correlated with increased cell‒cell connections influenced by high temperature adaptation. The expression of chemotaxis genes such as methyl-accepting chemotaxis protein (*mcp*) and chemotaxis phosphatase (*cheZ*) were also seen during the summer conditions which supported different processes of signal transduction. The *mcp* gene is a common receptor in prokaryotes that can sense chemical cues to relocate towards more favourable microhabitats (Salah Ud-Din and Roujeinikova 2017). The differential expression of this gene under summer conditions can play an important role in the signal transduction (Hickman et al. 2005), flagellum biosynthesis (Berleman and Bauer 2005), and exopolysaccharide biosynthesis (Black and Yang 2004). The *cheZ* plays a key role in the two-component regulatory system by catalysing the dephosphorylation of the response regulator CheY, and is critical for rapid response of prokaryotes to environmental stimuli (Zhao et al. 2002). The presence of pili was also supported at the gene level with differential expression of *pilY1*, a type IV pili, which enable prokaryotic adherence, motility, DNA uptake, and microcolony formation (Craig et al. 2019).

Recent studies on *Salmonella* suggest that lipid A remodelling plays an important role in the biogenesis of membrane vesicles (Bonnington and Kuehn 2016, Elhenawy et al. 2016). In agreement with this study, our analysis revealed that the genes *waaF* and *rfaF* play important roles in the biosynthesis of lipopolysaccharides (LPSs) required for vesicle biogenesis during summer conditions (Fig. 5). Our scanning electron micrographs did not show any cells with flagella, however, a number of genes responsible for flagella biosynthesis such as *fliD, fliE, fliG, flip, flhB, flrB, fleS*, and *fleL* were more abundant under summer conditions than winter conditions (Fig. 5). This observation matches well with that of Lauro et al. (2009), who reported the presence of cell motility genes in copiotrophs compared to oligotrophic marine strains, suggesting a copiotrophic response in the summer treatment. Although the presence of flagellin genes under summer conditions suggested the potential existence of flagellated cells, further scrutiny of scanning electron micrographs is necessary to confirm the observation of such cells. However, the presence of flagellin filaments along with vesicles at the end of the experiment indicated the possibility of flagellated cells during the summer conditions (Supplementary Figure S8D–F). Overall, motility is dependent on the season and availability of nutrients in natural aquatic ecosystems (Mitchell et al. 1995, Mitchell and Kogure 2006).

In addition to morphological genes, several genes with potential role in defence mechanisms, stress response, and regulation of ionic gradients were found to be differentially abundant. A few genes such as *dpo* and *xtmB*, related to the bacteriophage life cycle was also identified under summer conditions (Supplementary Table S5). The *dpo* gene encodes phage-encoded depolymerases, which degrade bacterial surface polysaccharides, facilitating phage infection (Knecht et al. 2020). The *xtmB* gene encodes the large subunit of the phage terminase enzyme, which is crucial for packaging phage DNA into the capsid during the assembly of new virions, driven by adenosine triphosphate (ATP)-dependent translocation (Wangchuk et al. 2021). The presence of these phage-related genes in conjunction with the two prokaryotic defence genes *hsdM* and *K07504* in summer suggested co-occurrence of both phage infection and prokaryotic defence mechanisms. The *hsdM* gene encodes the modification subunit of *hsd* (host specificity for DNA) and is part of Type I DNA restriction–modification system for protecting host cell from foreign DNA (Cooper et al. 2017). Additionally K07504 belong to type IV class of restriction–modification system, which also plays a role in defence mechanisms (Lepikhov et al. 2001). Various stress response genes, *rpoE, rpoS*, and *rpoN* were found, suggesting a potential role in responding to nutrient deprivation, temperature stress, toxicity, pH changes, and oxidative stress (Battesti et al. 2011, Mitchell and Silhavy 2019). Moreover, five genes of Na^+^-transporting NADH: ubiquinone oxidoreductase (*nqrB, nqrC, nqrD, nqrE*, and *nqrF*) were identified, indicating their importance in osmoregulation (Reyes-Prieto et al. 2014), which suggested that low maintenance respiration under the summer conditions does not necessarily indicate the absence of the processes.

### Linking maintenance activities to genes during winter conditions

According to the power law approximation, Kempes et al. (2017) reported that protein repair, regulation of ion gradients, RNA and ribosome repair are energy demanding maintenance activities. In our analysis, we identified processes associated with regulation of ion gradients and translation similar to Kempes et al. (2017), as well as signal transduction and energy metabolism, all potentially involved in maintenance activities under winter conditions (Fig. 4). The abundance of the signal transduction category indicated that prokaryotes coordinate their cellular responses to environmental changes through regulatory pathways, such as two-component systems, allowing adaptation and survival under various stress conditions (Hussa et al. 2007, Kojetin et al. 2007). Energy metabolism was a major metabolic pathway during low productivity, thereby probably related to maintenance activities. Additionally, the upregulation of translation category suggested a role in ribosome biogenesis and translation supporting maintenance activities related to cold adaptation by increased ribosome abundance per cell (Fig. 4). This upregulation indicated that prokaryotes, during periods of high maintenance activity, may allocate energy to produce new ribosomes, thereby enhancing the synthesis rate of specific proteins necessary for maintenance.

The analysis of differential gene expression revealed the abundance of different transporters, nitrogen metabolism, osmoregulation, ribosome biogenesis, and cold stress under winter conditions (Fig. 5, Supplementary Table S5). The identified transporters, including urea-proton symporter and ammonium transporters, highlight the crucial role of nitrogen availability in the nitrogen-limited marine ecosystems. Being a part of the structural components of nucleic acids and proteins, nitrogen is one of the basic elements for the survival and maintenance of prokaryotes. The abundance of these transporters might be advantageous during the high maintenance activities. The presence of reduced nitrogen in the form of urea and ammonium represents >50% of total nitrogen in the marine environments (Biller et al. 2015). Prokaryotes in nitrogen-limited ocean regions possess various urea transporters (Wang et al. 2023), while transporters from the Amt/Mep family facilitate the uptake of reduced nitrogen for biosynthesis and energy metabolism (Andrade and Einsle 2007). The efficient uptake of reduced nitrogen by these specific transporters is critical for the general maintenance and survival of prokaryotic cells (Andrade and Einsle 2007). The abundance of beta subunits of nitrate reductase (*nar*) and nitrite oxidoreductase (*nxr*) during winter conditions also supported the respective nitrate reduction and nitrite oxidation processes in marine bacteria (Maza-Márquez et al. 2022). These different processes are crucial steps in nitrogen cycling, contributing to energy supply during winter when access top organic carbon is reduced.

Maintaining a solute gradient is another essential maintenance process that demands a substantial amount of energy and thereby respiration on thermodynamic grounds (Neijssel and Tempest 1976). Accordingly, the increased abundance of the osmoregulatory gene TC.SSS, a member of the solute symporter (SSS) family, supports the cotransport of various solutes including sugars, amino acids, and vitamins, along with sodium ions across the membrane. This mechanism utilize the sodium gradient for nutrient uptake and environmental adaptation (Henriquez et al. 2021). Additionally, there were abundance of genes with an important role in the ribosome biogenesis, which was relevant to produce specific ribosomal subunits (Supplementary Table S5). We assume that these abundant ribosomal proteins under winter conditions likely play a crucial role in catalysing specific proteins necessary for driving maintenance activities, although the precise roles of these specific proteins remain to be elucidated. Furthermore, genes associated with cold stress were also abundant, aligning with earlier reports that emphasize their relevance for the survival and maintenance of marine prokaryotes in cold environments (Nakaminami et al. 2006).

It has been reported that prokaryotic cell-specific respiration rates are greater at low temperatures and low specific growth rates (Vikström and Wikner 2019, Verma et al. 2022). The same phenomenon of higher respiration at low temperature has also been reported in yeast (Römisch and Matheson 2003), fish (Johnston and Dunn 1987), and plants (Talts et al. 2004). The presence of high specific respiration rates at low specific growth rates primarliy supports maintenance activities. We speculate that elevated specific respiration may drive the synthesis of a higher cellular adenylate pool, including ATP and adenosine diphosphate (ADP). It has been shown experimentally in *Psychrobacter* that a decrease in temperature (−5°C) leads to a significant increase in the concentration of the adenylate pool, suggesting an adaptation strategy to maintain reaction rates at low temperature (Amato and Christner 2009). In our analysis, we could not find any differential expression of ATPase under winter conditions to support this hypothesis, however a total of 49 non-DE ATPase genes were present during both winter and summer conditions (Supplementary Table S5). This indicated that large temperature differences involving freezing temperature might be required to detect significant changes as shown earlier by Amato and Christner (2009). Also, the quantitative estimation of the cellular adenylate pool can provide a better explanation of their role and significance in the maintenance activities. This will help to better understand how marine prokaryotes adjust their energy requirements between low and high productive marine ecosystems. We could not detect any significant gene expression changes related to the adenylate pool, however the higher abundance of KEGG category, i.e. ‘Energy metabolism’ under winter conditions highlighted the role of pathways supporting energy expenditure for driving various maintenance activities.

## Conclusion

In conclusion, our study examined how prokaryotic communities adapt in terms of morphology and maintenance activities under simulated winter (low productivity) and summer (high productivity) conditions at the mesocosm scale. Under winter conditions, prokaryotes did not exhibit distinct morphological features, likely due to limited energy and productivity levels in such environments. However, their metabolic activity indicated a focus on maintenance metabolism with genes related to nitrogen transport, osmoregulation and ribosome biogenesis playing key roles. In contrast, under the more productive summer conditions, prokaryotic communities showed distinct morphological features supported by differential gene transcription. Due to the presence of higher temperatures and nutrient levels under summer conditions, prokaryotes had higher specific growth rates, which favoured investment by the cells in the morphological features to optimize their ecological fitness. The differential expression of genes associated with signal transduction, stress response, transporters, phage infection, and prokaryotic defence mechanisms was observed, suggesting their potential role in maintenance activities. Maintenance activities also occurred during high productivity summer conditions albeit with low expression levels. Thus, the balance between maintenance and growth activities shift with the ecosystem’s productivity. Considering that most marine ecosystems have low productivity and are energy limited, further studies aimed at deciphering prokaryotic metabolic adaptations is motivated to expand our understanding of the ecological and evolutionary importance of maintenance activities.

## Supplementary Material

fiae121_Supplemental_Files

## Data Availability

The raw data for the different prokaryotic variables is available at the Pangaea repository: https://doi.org/10.1594/PANGAEA.956053. The electron micrographs from the scanning and transmission electron microscopy are deposited at https://doi.org/10.6084/m9.figshare.25285873.v3, and the primary data related to cell morphology, elemental composition and particles abundance is available at https://doi.org/10.6084/m9.figshare.25285948.v3. The metatranscriptomic sequences can be found in the European Nucleotide Archive (ENA) under accession number PRJEB63211. The code used to produce figures based on the metatranscriptomic data can be found at GitHub (https://github.com/dennisamne/PROMAC_morphology).
