# Supplementary material for: Prokaryotic morphological features and maintenance activities governed by seasonal productivity conditions
Source: FEMS Microbiol Ecol. 2024 Sep 11;100(11):fiae121. doi: 10.1093/femsec/fiae121 (PMC11556340; doi:10.1093/femsec/fiae121)
Supplement: fiae121_Supplemental_Files [file fiae121_supplemental_files.zip › MS2_Supplementary Data.docx]

# **Prokaryotic morphological features and maintenance activities governed by seasonal productivity conditions**

# **Supplementary Data**

**Supplementary Table S1**. Descriptive statistics of different prokaryotic variables in the C and TN treatment for Days 10 and 17 of the experiment. See Supplementary Figure S1 legend for abbreviation of variables. All the data have been taken from Verma et al. (2022) unless otherwise indicated. The average values and ± 2 × SE (n = 6) are shown.

| **Variable (units)** | **Treatment** | |
| --- | --- | --- |
|  | C | TN |
| Temperature (°C) | 0.7 ± 0.3 | 10.1 ± 0.3 |
| PA (cells dm^-3^) x 10^9^ | 1.17 ± 0.06 | 2.62 ± 1.18 |
| BioVol (µm^3^) | 0.03 ± 0.00 | 0.05 ± 0.01 |
| *µ* (d^-1^) | 0.14 ± 0.03 | 2.28 ± 0.57 |
| PG (cells dm^-3^ d^-1^) x 10^9^ | 0.16 ± 0.03 | 6.37 ± 2.97 |
| PR (µmol O_2_ dm^-3^ d^-1^) | 2.15 ± 1.45 | 8.20 ± 3.95 |
| *ρ* (fmol O_2_ cell^-1^ d^-1^) | 1.84 ± 1.34 | 3.62 ± 0.87 |
| *ρ_m_/ρ* (%) | 75 ± 4.10 | 16 ± 4.20 |
| Dissolved organic carbon, DOC (µmol dm^-3^) | 368.1 ± 17.3 | 347.9 ± 7.2 |
| Total dissolved nitrogen, TDN (µmol dm^-3^) | 18.4 ± 0.1 | 18.0 ± 2.3 |
| Total dissolved phosphorus, TDP (µmol dm^-3^) | 0.2 ± 0.0 | 0.2 ± 0.1 |
| Relative atomic %, C/Cr^¥^ | 2.36 ± 0.33 | 2.47 ± 0.35 |
| Relative atomic %, N/Cr^¥^ | 0.28 ± 0.07 | 0.27 ± 0.06 |
| Relative atomic %, P/Cr^¥^ | 0.03 ± 0.01 | 0.04 ± 0.01 |
| C: N: P (relative to P) ^¥^ | 85:10:1 | 63:7:1 |
| Particles concentration (*µ*g/mL) ^¥*^ | 9.34 ± 0.47 | 38.3 ± 3.00 |

*Values measured at the end of the experiment.

^¥^Data generated in the present study.

**Supplementary Table S2**. Median percentage distribution of cell shapes analysed through epifluorescence microscopy (EpiM) and scanning electron microscopy (SEM) in the C and TN treatments. The *n* value in the parentheses shows the number of cells analysed in each category. The values show median ± 2 × SE (n = 6).

| **Cell shape** | **Microscopy** | **Treatment** | |
| --- | --- | --- | --- |
|  |  | C (*n*) | TN (*n*) |
| Rod | EpiM | 83.5 ± 1.9 (5687) | 67.5 ± 7.2 (6388) |
|  | SEM | 67.8 ± 6.9 (126) | 40.6 ± 10.3 (127) |
| Vibrioid | EpiM | 16.5 ± 1.9 (1165) | 32.5 ± 7.2 (2775) |
|  | SEM | 32.2 ± 6.9 (61) | 59.4 ± 10.3 (172) |

**Supplementary Table S3**. Combinations of different prokaryotic morphological features in the C and TN treatment based on SEM method. See Supplementary Figure S2 for description of morphological features and Table 1 for description of odds ratio in main text.

| **Cell shape** | **Combinations of features** | **Chi-square, p-value** | **Odds ratio (TN/C)** |
| --- | --- | --- | --- |
| Rod | Connections + Extracellular + Pili | 3.6, 0.05 | 6.2 |
| Vibrioid | Connections + Pili | 3.6, 0.05 | 4.0 |
|  | Connections + Blebbing | 9.7, 0.002 | 13.2 |
| Rod + Vibrioid | Connections + Pili | 4.8, 0.03 | 2.0 |
|  | Connections + Blebbing | 20.9, < 0.001 | 13.6 |

**Supplementary Table S4**. Metatranscriptomic sequencing statistics in the C and TN treatment

Provided in a separate Excel file.

**Supplementary Table S5A**. List of differentially expressed genes in the C and TN treatment based on KEGG database

**Supplementary Table S5B.** List of non-differentially expressed (non-DE) genes in the C and TN treatment based on KEGG database

**Supplementary Table S5C.** List of differentially expressed morphology and maintenance activity genes in the C and TN treatment based on KEGG database

Provided in three worksheets of a separate Excel file.

**Supplementary Table S6**. Maintenance activity genes and morphology gene list compiled from the literature

Provided in a separate Excel file.

**Supplementary Table S7**. Maintenance and morphological gene transcripts differentially abundant in the C and TN treatment analysed from a priori list (see Supplementary Table S6). The classification of different gene transcripts along with categories and descriptions based on the KEGG database are shown. KEGG: Kyoto Encyclopedia of Genes and Genomes.

| **Treatment** | **Gene** | **KEGG KO** | **Category** | **Sub-category** | **Description** |
| --- | --- | --- | --- | --- | --- |
| C | narY | K00371 | Metabolism | Signal transduction | nitrate reductase / nitrite oxidoreductase, beta subunit [EC:1.7.5.1 1.7.99.-] |
| TN | dps | K04047 | Oxidative stress | Resistance to environmental stress | starvation-inducible DNA-binding protein |
|  | hsdM | K03427 | Defence mechanism | Type I restriction | type I restriction enzyme M protein [EC:2.1.1.72] |
|  | poxB | K16243 | Metabolism | Xenobiotic biodegradation | phenol/toluene 2-monooxygenase (NADH) P1/A1 [EC:1.14.13.244 1.14.13.243] |
|  | rfaE | K03272 | Metabolism | Lipopolysaccharide biosynthesis | D-beta-D-heptose 7-phosphate kinase / D-beta-D-heptose 1-phosphate adenosyltransferase [EC:2.7.1.167 2.7.7.70] |


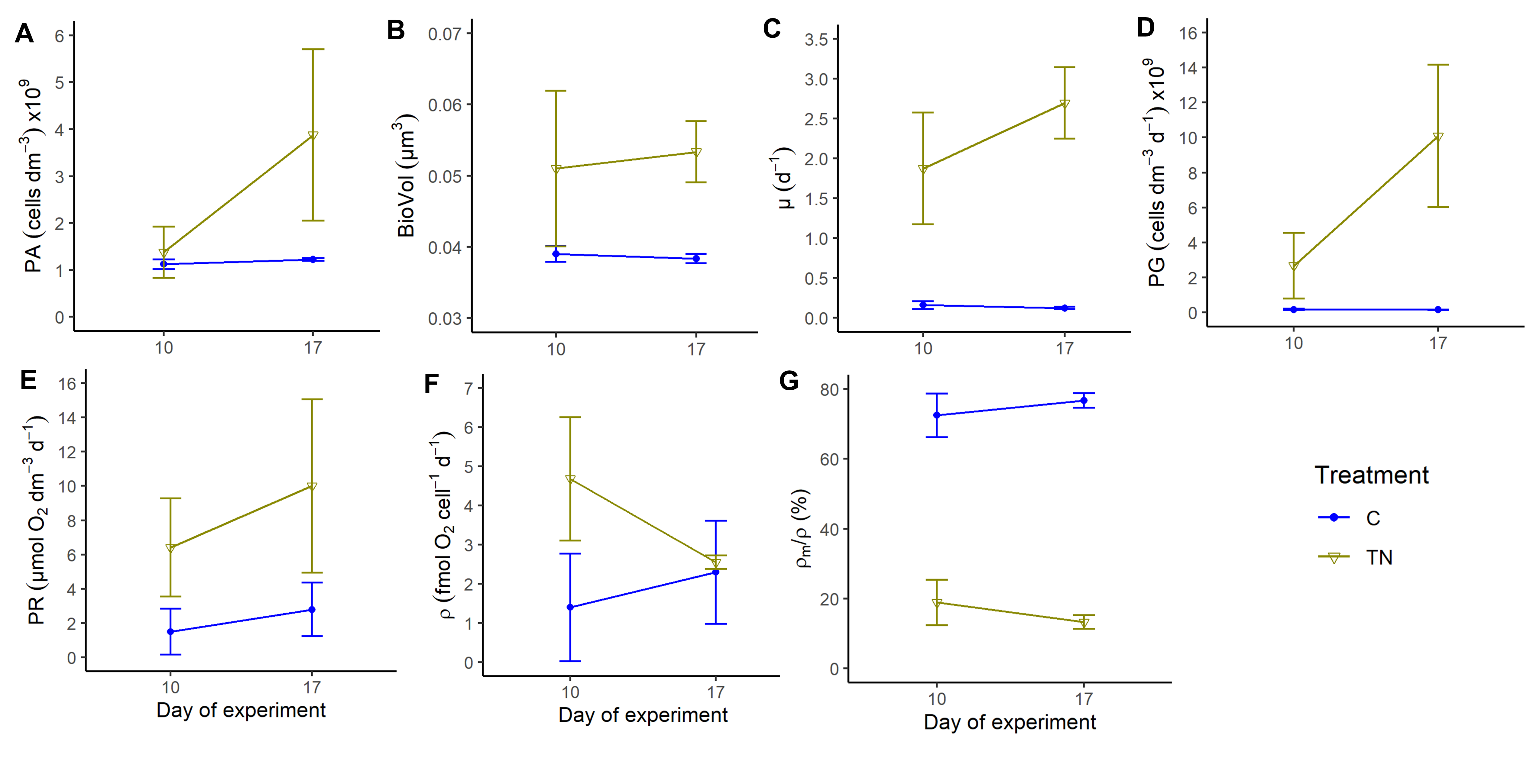


**Supplementary Figure S1**. Patterns of prokaryotic variables in the C and TN treatment for Days 10 and 17 of the experiment. A: PA, prokaryotic abundance; B: BioVol, prokaryotic cell biovolume; C: *µ*, prokaryotic specific growth rate; D: PG, prokaryotic growth; E: PR, prokaryotic respiration; F: *ρ*, specific prokaryotic respiration; G: *ρ_m_/ρ*, prokaryotic maintenance respiration. Error bar shows ± 2 × SE (n = 6).


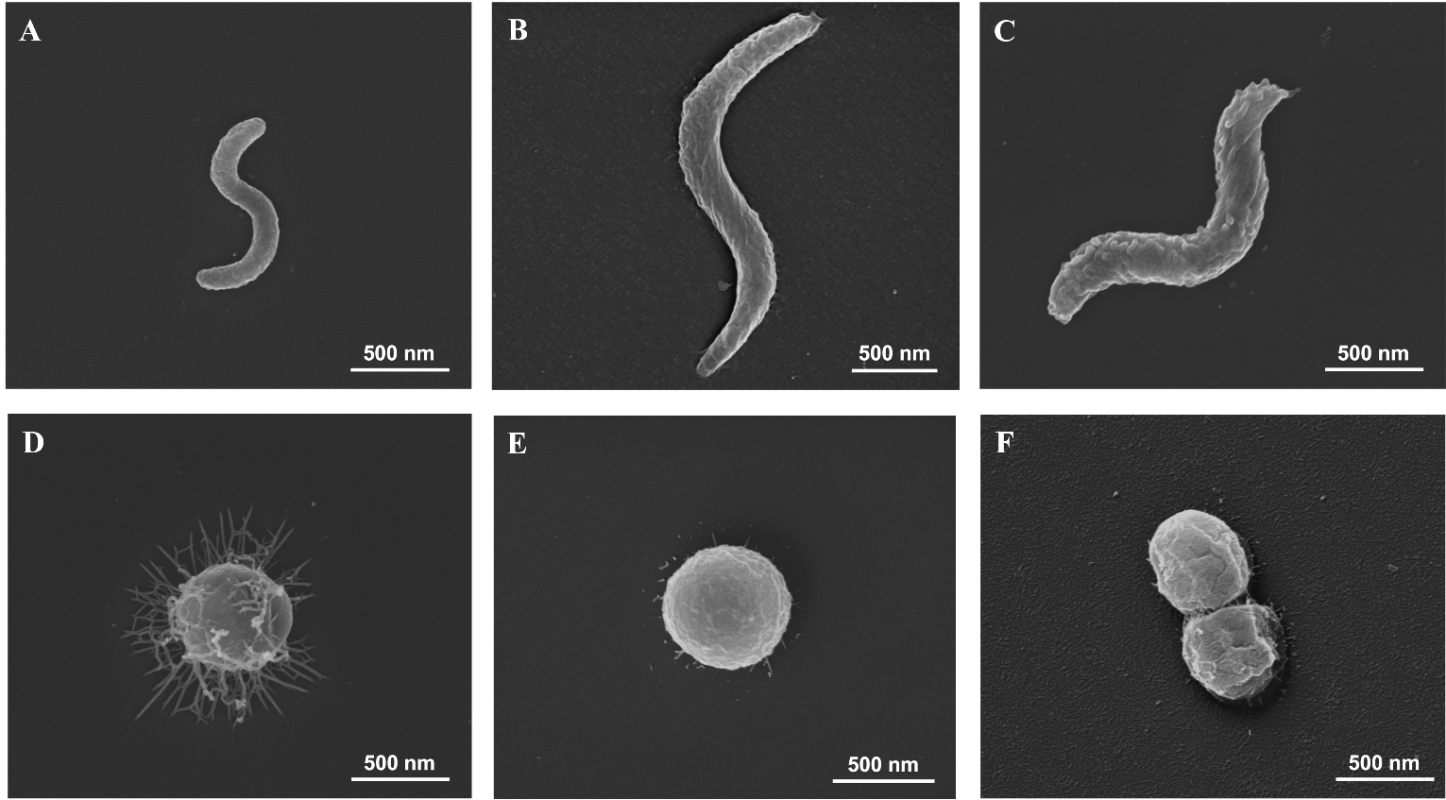


**Supplementary Figure S2**. Prokaryotic cell shapes in the C and TN treatments except rods and vibrioids based on SEM. A-C: spiral cell shapes; D-F: coccoid cell shapes. Scale bar, 500 nm.

**
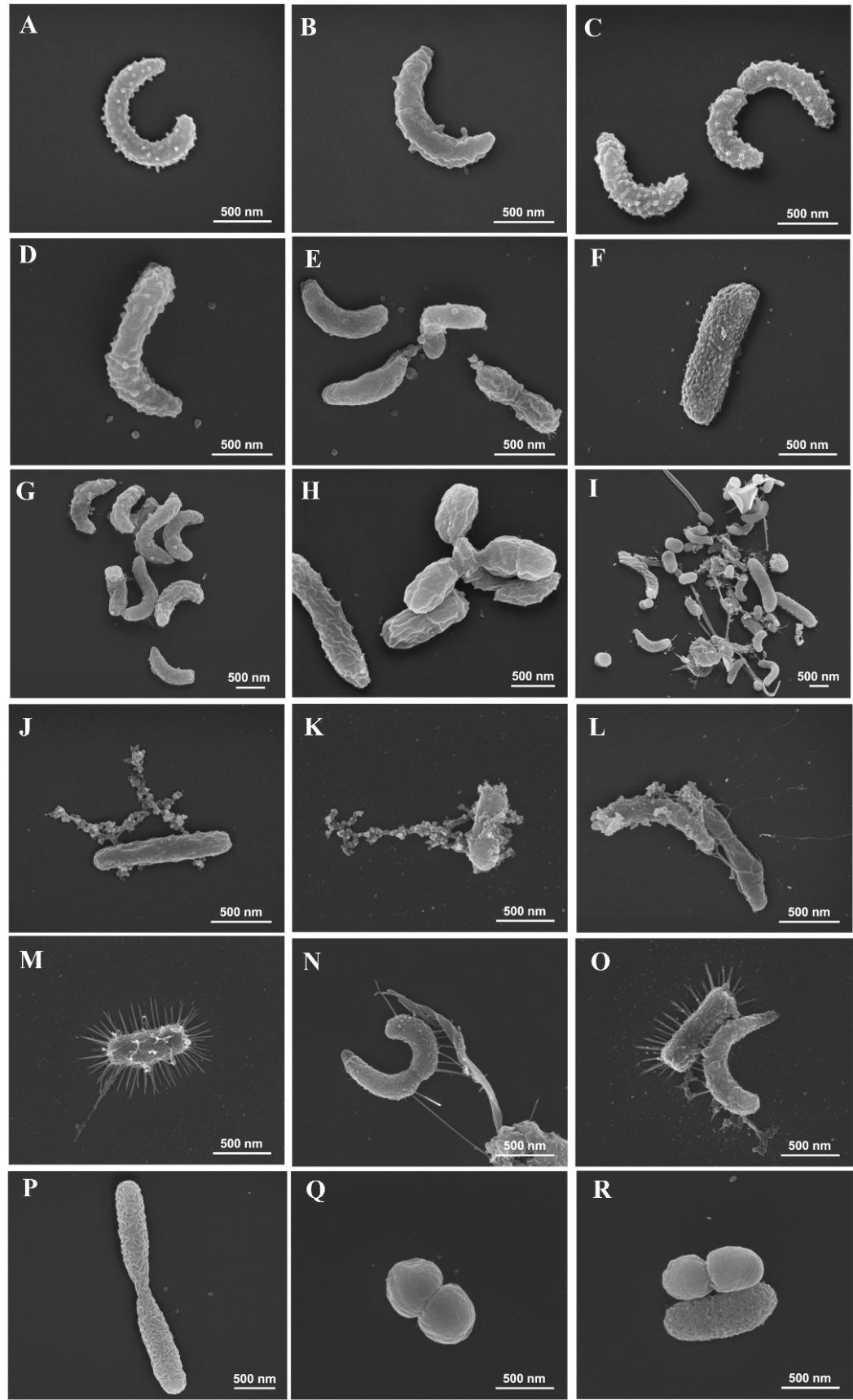
**

**Supplementary Figure S3**. Representative prokaryotic morphological features in the C and TN treatment based on SEM. A-C, membrane blebbing; D-F, membrane vesicles; G-I, cell‒cell connections; J-L, extracellular polymeric substances (EPS); M-O, pili; P-R, cell division. Scale bar, 500 nm.


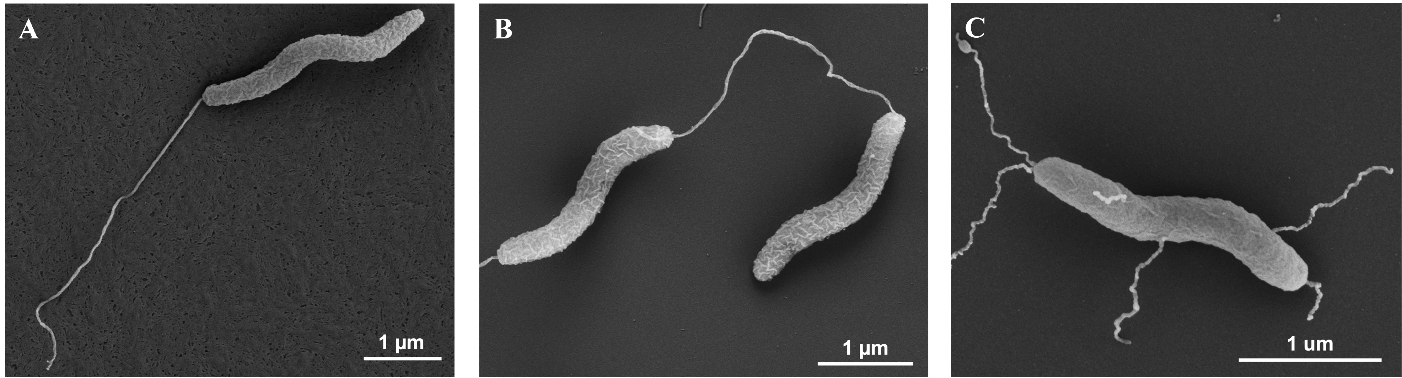


**Supplementary Figure S4**. Scanning electron micrograph of a marine bacterium *Marinomonas* sp. GOBB3-320 with flagella (A-C). Scale bar, 1 µm.


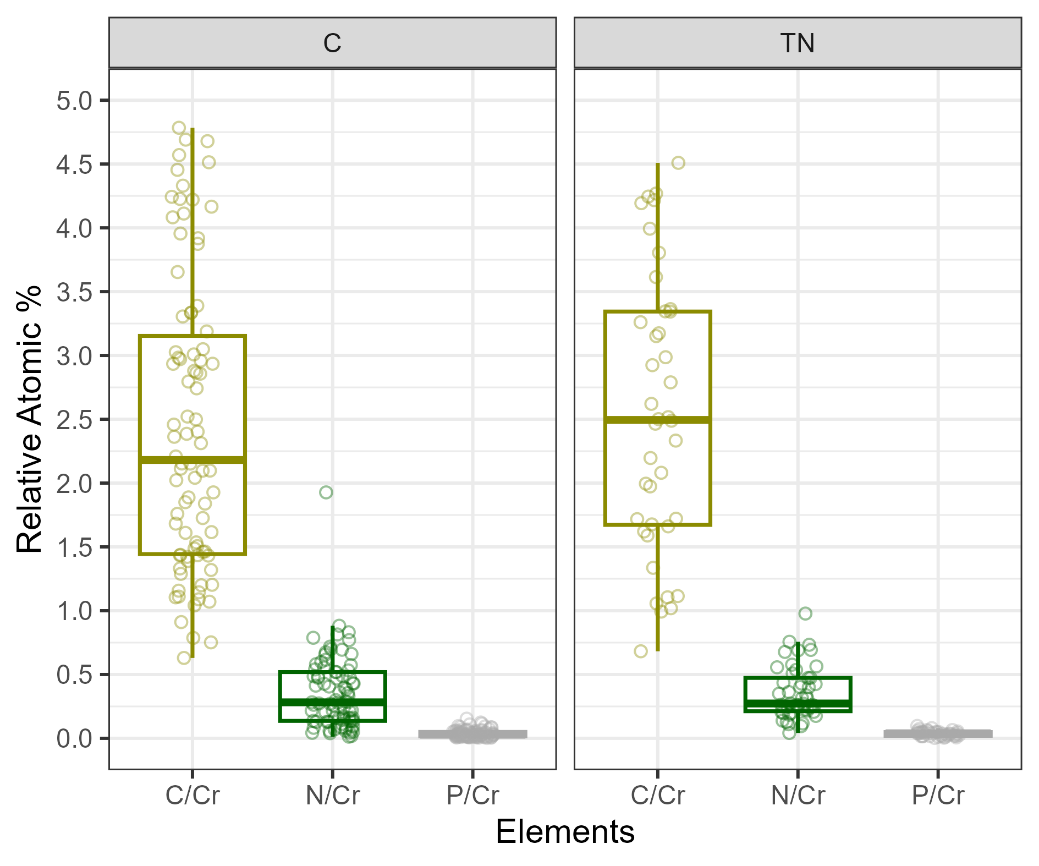


**Supplementary Figure S5**. Elemental composition of prokaryotes in the C and TN treatment. The x-axis shows different elements: carbon (C), nitrogen (N), and phosphorus (P) in relation to chromium (Cr) and the y-axis shows the relative atomic % of these elements in comparison to total elements in the cell.


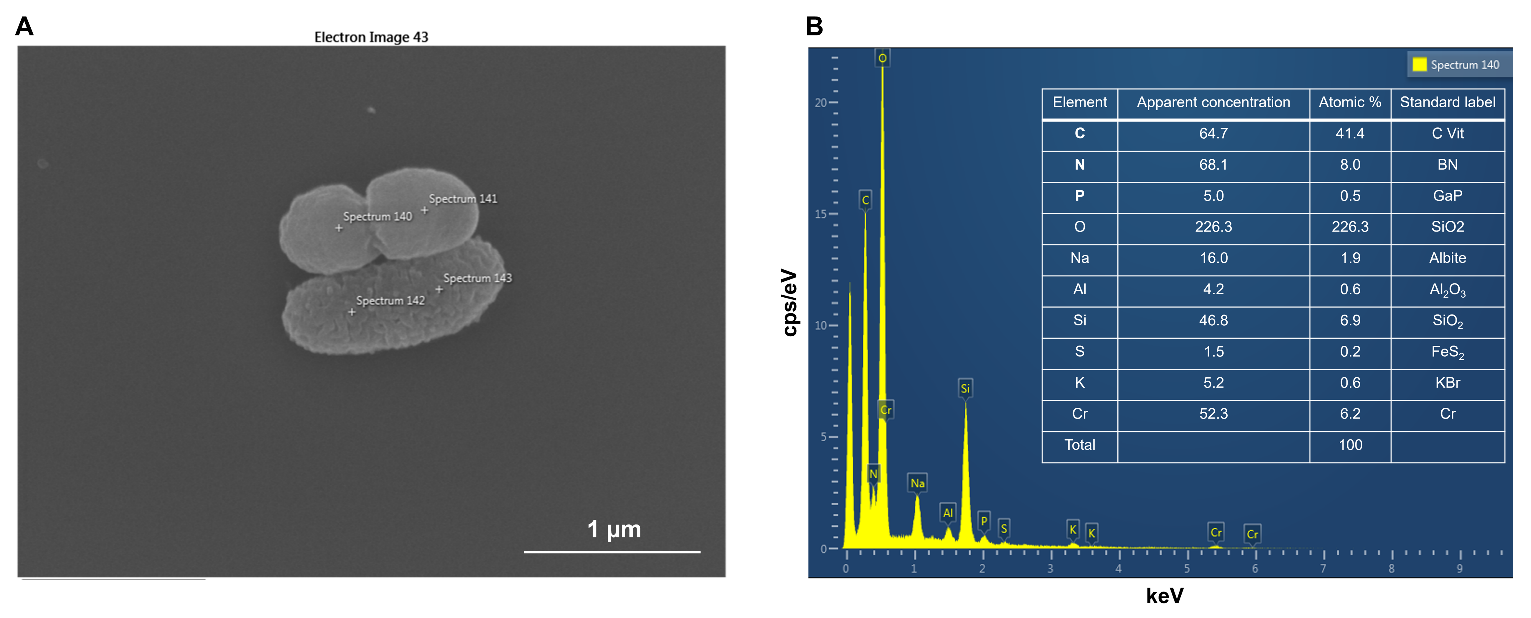


**Supplementary Figure S6**. Prokaryotic morphology in SEM and the coupled SEM-EDX analysis for one of the analysed spectra. The panel A shows two connected prokaryotic cells with two spectra each. Scale bar, 1 µm. A background spectrum was taken to correct the sample spectrum (not shown). The panel B shows the elemental profile of spectrum 140 and the inset table shows the atomic % of different elements. The x-axis shows the accelerating voltage range in keV (kilo-electron volt) and the y-axis shows the counts per second per electron-volt (cps/eV).


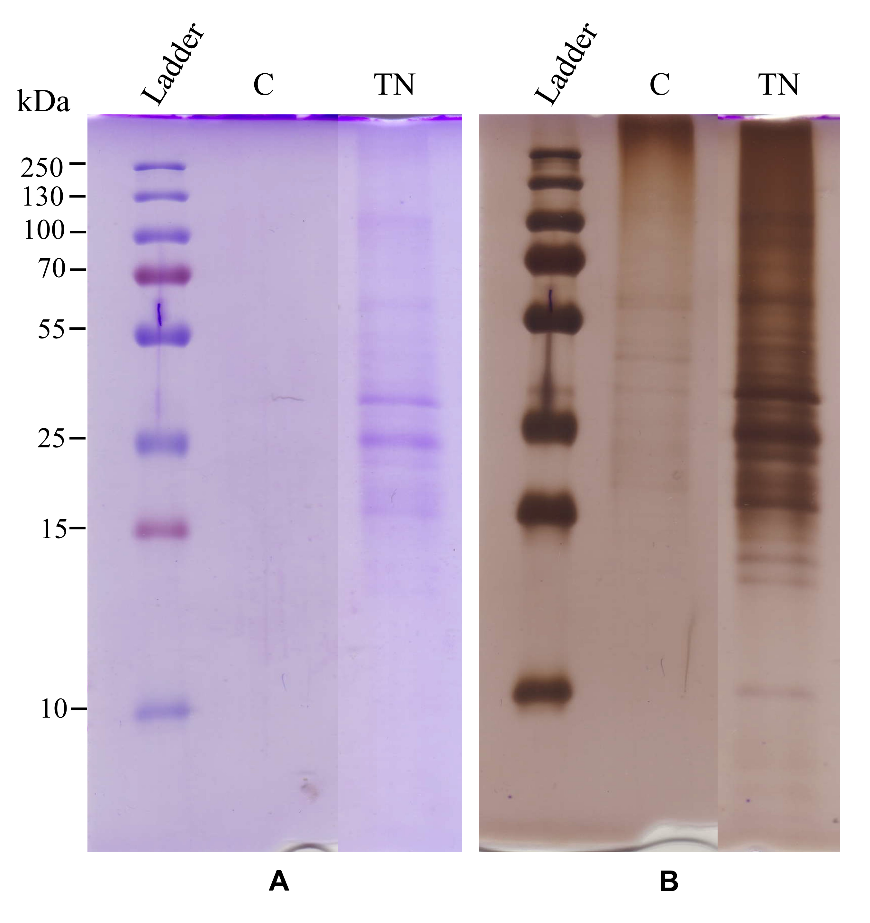


**Supplementary Figure S7.** SDS-PAGE gel of crude fraction of extracted vesicles in the C and TN treatment. A, Coomassie-brilliant-blue stained gel. B, silver staining of the Coomassie-brilliant-blue stained gel. Ladder: Protein standard ladder, 10-250 kDa.


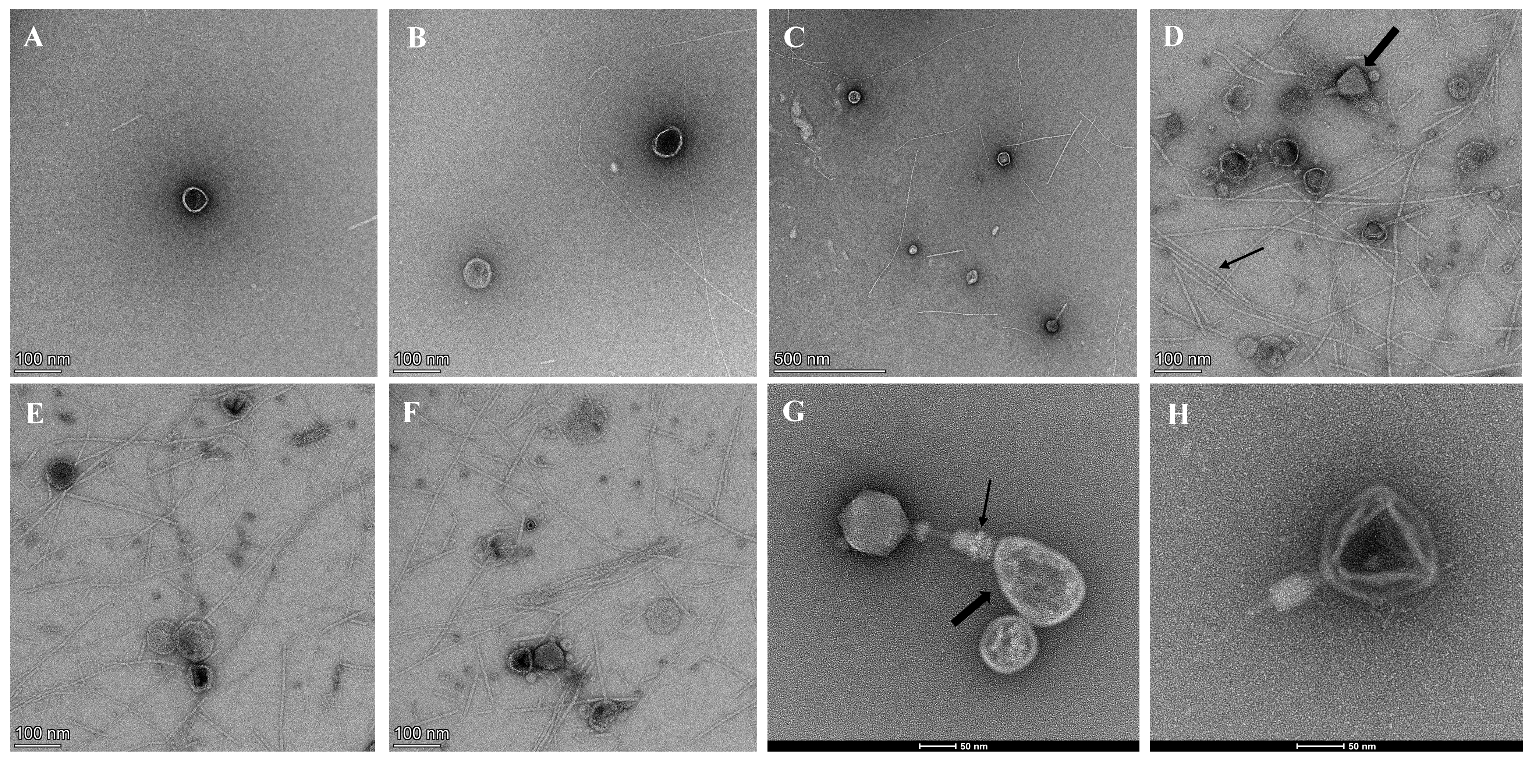


**Supplementary Figure S8**. Transmission electron microscopy (TEM) of the crude fraction of extracted vesicles in the C and TN treatment. A-C, membrane vesicles in the C treatment. D-F, membrane vesicles, phages and flagellin filaments in the TN treatment. A thin and thick black arrow shows flagellin filaments and a phage respectively in the D panel. G, interaction of a phage with the membrane vesicle in the TN treatment. The thin and thick black arrow shows the tail of a phage and membrane vesicle respectively in the G panel. H, phage with a capsid and a tail in the TN treatment. Scale bar, 100 nm (in panels A-F, except panel C where scale bar is 500 nm). Scale bar, 50 nm (in panels G and H).


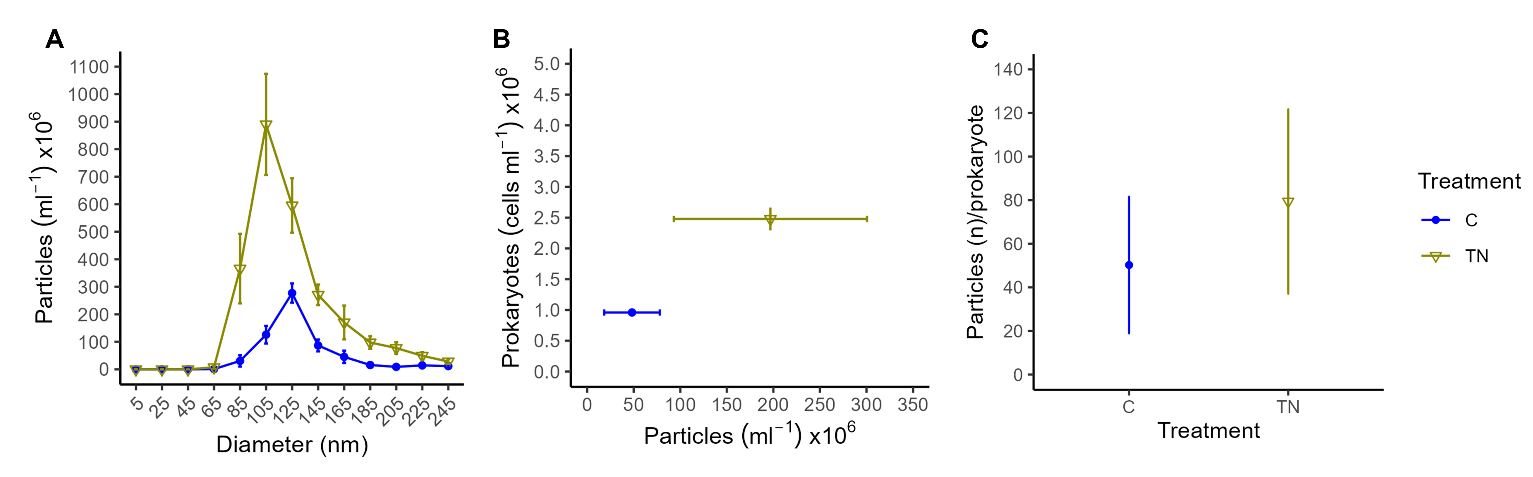


**Supplementary Figure S9**. Abundance and size distribution of particles (phages and membrane vesicles) and prokaryotes in the C and TN treatment. (A) Particle size distribution in the crude fraction of vesicles. x-axis shows the particle diameter in nm and the y-axis shows their abundance. Five videos were used to calculate the average abundance of particles with corresponding diameter. (B) Abundance scatterplot between particles (x-axis) and prokaryotes (y-axis). (C) Median number of particles (n) in relation to a prokaryote. Error bar shows ± 2 × SE in all panels.


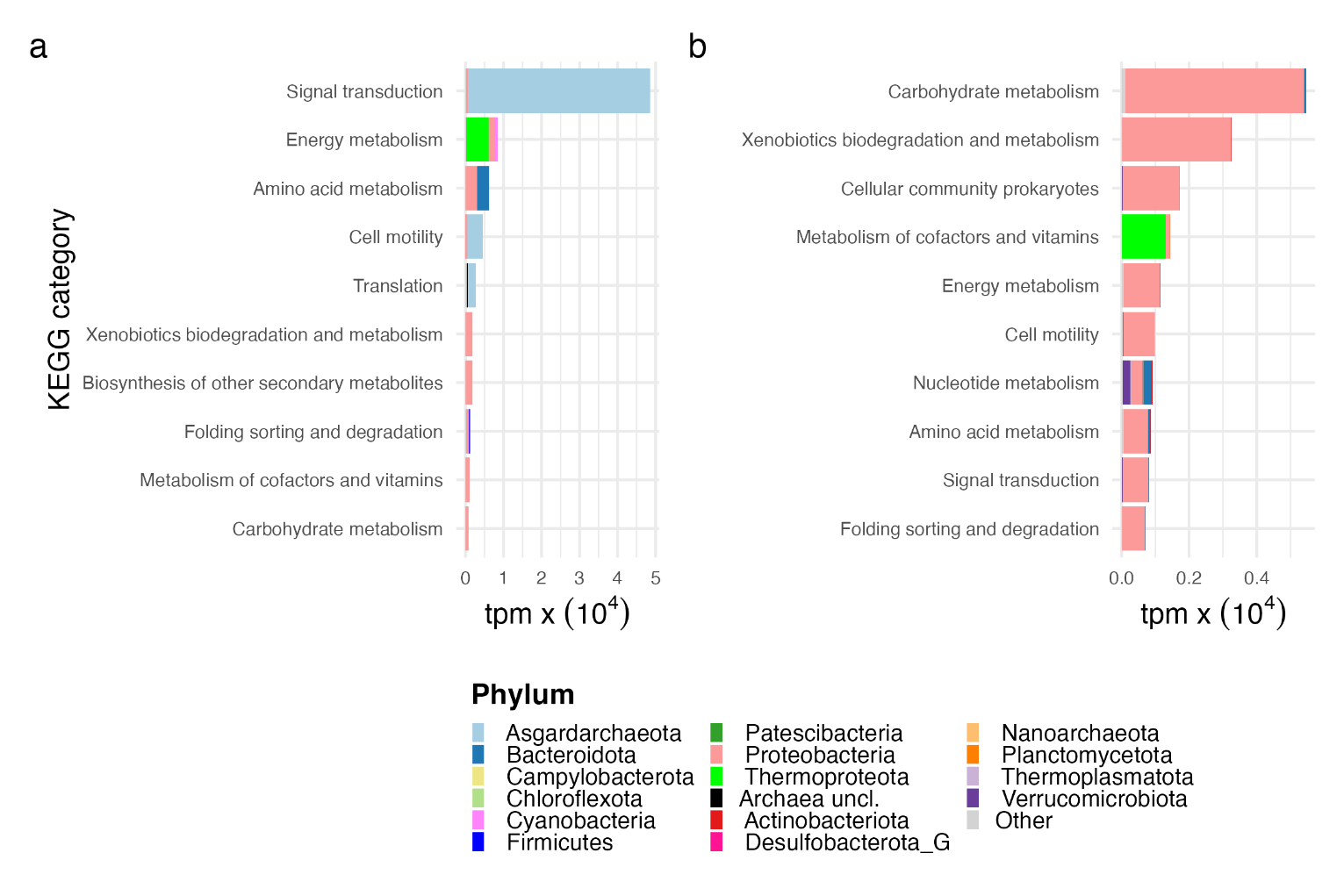


**Supplementary Figure S10**. Differentially abundant Kyoto Encyclopedia of Genes and Genomes (KEGG) categories as a function of transcripts per million (tpm) in the C (a) and TN treatment (b). Different colour shadings in the barplot represent taxonomy at the phyla level.
